# Neural correlates of schema-dependent episodic memory and association with behavioral flexibility in autism spectrum disorders and typical development

**DOI:** 10.1186/s11689-021-09388-9

**Published:** 2021-09-15

**Authors:** Kevin M. Cook, Xiaozhen You, Joseph Bradley Cherry, Junaid S. Merchant, Mary Skapek, Meredith D. Powers, Cara E. Pugliese, Lauren Kenworthy, Chandan J. Vaidya

**Affiliations:** 1 Interdisciplinary Program in Neuroscience, Georgetown University, 401 White-Gravenor, 37th and O Streets NW, Washington, DC 20007 USA; 2 Children’s Research Institute, Children’s National Health System, Washington, DC 20010 USA; 3 Department of Psychology, University of Maryland, College Park, MD 20742 USA; 4 University of Connecticut, Storrs, CT 06269 USA; 5 University of Virginia, Charlottesville, VA 22904 USA; 6 Department of Psychology, Georgetown University, Washington, DC 20007 USA

**Keywords:** Behavioral flexibility; Medial temporal lobe, fMRI, Associative memory, Prefrontal cortex, Executive function

## Abstract

**Background:**

Conceptual knowledge frameworks termed *schemas* facilitate memory formation and are posited to support flexible behavior. In adults, the medial temporal lobe (MTL) and medial prefrontal cortex (mPFC) trade-off in supporting schema-based memory formation, such that encoding of subsequently remembered schema-congruent information relies on mPFC, whereas schema-incongruent information relies on MTL. Whether this is true in the immature brain and relates to behavioral flexibility is unknown. In this preliminary investigation, we aimed to replicate the adult findings in typically developing (TD) children and to investigate the relevance to behavioral flexibility by examining a disorder with pathognomonic behavioral rigidity, autism spectrum disorder (ASD).

**Methods:**

Children completed an associative subsequent memory paradigm, encoding object-scene pairs in an MRI scanner and subsequently completing a recognition test outside the scanner after a delay. Recognition performance was back sorted to construct remembered vs forgotten contrasts. One-way ANOVAS were conducted in MTL and mPFC masks for schema-congruency, followed by congruency by flexibility scores. Exploratory analyses were then conducted within the whole brain.

**Results:**

As reported in adults, episodic memory was strongest for schema-congruent object-scene pairs, followed by intermediate pairs, and lowest for schema-incongruent pairs in both TD and ASD groups. However, the trade-off between mPFC and MTL in TD children differed from adult reports such that mPFC supported memory for intermediate schema-congruency and left anterior MTL supported memory for schema-congruent pairs. In ASD, mPFC engagement interacted with flexibility such that activation supporting memory for intermediate schema-congruency varied with parent-reported flexibility and was higher in those with more flexible behavior. A similar interaction was also observed in both the left dorsolateral and rostrolateral PFC in whole-brain analysis.

**Conclusion:**

Our findings provide the first preliminary evidence for the association of schema-based episodic memory formation and behavioral flexibility, an executive function impaired in multiple developmental disorders. Upon replication, this line of research holds promise for memory-based interventions addressing executive problems of behavioral rigidity.

**Supplementary Information:**

The online version contains supplementary material available at 10.1186/s11689-021-09388-9.

## Background

Integration of new experiences into prior knowledge serves to facilitate memory and promote flexible behavior in everyday life. One example of such integration is represented by *schemas*, which are conceptual knowledge frameworks formed from extracting common elements shared across similar experiences. Schemas scaffold the acquisition of new knowledge and promote flexible adaptation to the environment by enabling generalization to new experiences [67, 70, 89]. Studies in adults show that information that is congruent with schemas is recognized and recalled more accurately [7, 12, 78], and faster [55] than incongruent information. Further, typically developing (TD) children as young as 3–5 years old exhibit better recall for events consistent with schemas for autobiographical information (e.g., pediatrician visits, [9, 54]), social and academic knowledge [21], and gender stereotypes [8, 83]. Children also exhibit greater false recollection for schema-congruent events [33, 53]. Thus, the formation and use of schemas starts early in development and is posited to aid in flexible adaptation to the environment [26, 87, 89]. However, whether schema-based memory is associated with behavioral flexibility remains untested.

Here, we investigate the association between schema-based memory and behavioral flexibility by capitalizing on autism spectrum disorder (ASD), a neurodevelopmental condition that is marked by behavioral inflexibility. Support for this association would lay the foundation for delineating mechanisms by which abstract knowledge systems contribute to executive function. It would open up a new area of intervention targets for characteristics of behavioral inflexibility in ASD such as repetitive and rigid engagement in restricted sets of behaviors and interests [1]. Many children with ASD prefer strict adherence to schedules and routines [39] and have greater difficulty switching between tasks [58, 86]. Moreover, behavioral inflexibility is associated with the two core characteristics of ASD, difficulty in social interaction [25] and engagement in restricted and repetitive behaviors [42, 45], and also predicts socially adaptive outcomes [10, 59, 60], although the direct link between cognitive and behavioral flexibility remains tentative [25]. Behavioral studies in adults and children with ASD suggest a lack of reliance on schemas during memory performance across a wide range of contexts. While overall memory performance did not differ relative to TD, individuals with ASD remembered schema-congruent and incongruent elements equally on tests of word recall [24], narrative cued recall [48], and recognition of scene elements [24, 48, 49, 51]. Further, difficulties with prototype formation [38] and generalization of category knowledge [23] also suggest problems with schematic processing in ASD. To date, these two literatures, schematic memory and problems with flexibility, have remained separate in the study of ASD. An empirical linkage between them is necessary to adopt a unifying conceptual framework, which posits that memory integration into abstract knowledge contributes to flexible behavior. The purpose of the present study is to provide preliminary empirical support for that connection.

The neural basis of schema use has been sufficiently delineated in studies with adults, which provides a basis to formulate testable hypotheses in TD and in ASD. Studies with TD adults indicate that schema-dependent encoding relies on the reciprocal relationship between the medial prefrontal cortex (mPFC) and medial temporal lobe (MTL) [73, 76, 77]. Arbitrary associations between stimuli that are not congruent with pre-existing schema knowledge are primarily encoded in the hippocampus, whereas the encoding of greater schema-congruency relies on recruitment of the mPFC [44, 69, 78]. This mPFC-MTL gradient of schema-congruency was characterized by van Kesteren et al. [75] using a subsequent memory paradigm for scene-object pairings that were schema-congruent, schema-intermediate, and schema-incongruent. Specifically, mPFC and MTL traded off during encoding of subsequently remembered pairings, such that mPFC was engaged for congruent pairs while MTL was engaged for incongruent pairs; further, stronger functional connectivity between the two regions supported encoding of schema-congruency. Further work has suggested a posterior to anterior specificity axis within the MTL [32, 66, 68], with posterior hippocampus binding highly detailed and event-specific representations which progressively transition to gist representations anteriorly as a function of experience-based generalization [13]. Together, this work highlights the importance of MTL regions and mPFC in representing specific and schematic knowledge, respectively.

Two studies of typical development suggest that mPFC also supports immature schema-based memory. First, during retrieval of newly learned schemas (e.g., artificial animal categories), 8–12-year-old children engaged the mPFC, but to a lesser extent than adults [14]. MTL activation, however, did not differ between groups. Second, during encoding of subsequently remembered object-scene pairs, participants aged 6–7, 18–22, and 67–74 years engaged the mPFC to a greater extent for schema-congruent than incongruent pairs [15]; this study did not include children between 7 and 18 years or pairs with an intermediate level of schema-congruence. Nevertheless, it appears that similar to adults, mPFC supports schema-based memory formation in typical development.

The present study was motivated by three goals. First, we aimed to replicate in TD children the mPFC-MTL gradient supporting episodic memory formation varying in schema-congruency, as was observed in adults by van Kesteren et al. [75]. Using their stimuli and similar study design, we hypothesized selective recruitment of the mPFC and MTL based on schema-congruency of subsequently remembered object-scene image pairs, as classified by children’s subjective ratings of congruency with schematic knowledge (incongruent, intermediate, and congruent). Our study fills the gaps left by Brod and Shing’s [15] study, such as the inclusion of 8–15-year-old children, pairs of intermediate congruency, and subjective rather than canonical congruency classification during encoding. In light of the reviewed findings, we predicted that schema-congruency would facilitate object and associative recognition memory and engage mPFC whereas schema-incongruency would engage MTL. Second, we aimed to examine the neural correlates of schema-based episodic memory in children with ASD. Since past studies reported no advantage of schema-congruency in memory facilitation, we predicted that children with ASD would demonstrate neither schema-related memory facilitation nor schema-dependent brain activation. Third, we aimed to assess the putative relationship between schema-based memory and behavioral flexibility. Theoretical models reviewed earlier suggest that integrative memory processing serves adaptation to novel events, and thus, promotes flexible behavior. Such flexibility is impaired in autistic children to varying degrees, which can be measured by parental reports of their everyday behavior on questionnaires such as the Flexibility Scale [72]. The Flexibility Scale was designed to tap behavioral and social flexibility specifically in ASD, and therefore, it has limited sensitivity in TD children. Thus, we predicted that schema-based memory and mPFC engagement would be higher for more flexible children with ASD.

## Methods

### Participants

Nineteen TD children and 12 children diagnosed with ASD ranging in age from 8 to 15 years old (Table 1) participated in the study, complying with consenting guidelines for both Georgetown University and Children’s National Health System’s Institutional Review Boards. Two additional ASD and one TD child were recruited and excluded for performance and head motion reasons (see fMRI Processing). The TD children were recruited from the greater Washington, DC community and institutional research databases. The children with ASD were recruited through the Center for Autism Spectrum Disorders (CASD) at Children’s National Hospital in Washington, DC.

**Table 1:** Demographic characteristics

	Group	
	TD	ASD
*N* (*n* female)	19 (5)	12 (3)
Age (years)	11.77 (*2.51*)	13.18 (*1.73*)
IQ (standard score)	122.85 (*12.86*)	114.17 (*13.19*)
Maternal Ed (years)	18.21 (*3.29*)	16.50 (*3.45*)
Flexibility Scale* (raw score)	17.74 (*13.36*)	61.67 (*34.89*)
BRIEF BRI* (T-score)	50.38 (*25.42*)	77.50 (*37.67*)
BRIEF MI*	45.58 (*14.86*)	70.92 (*15.59*)
BRIEF GEC*	45.01 (*16.41*)	70.00 (*14.23*)
ADOS SA (raw)	-	9.72 (*3.72*)
ADOS SA (CSS)	-	7.54 (*2.34*)
ADOS RRB (raw)	-	2.73 (*1.35*)
ADOS RBR (CSS)	-	7.18 (*2.52*)
ADI-R Soc	-	20.33 *(5.48)*
ADI-R Comm	-	14.44 *(5.99)*
ADI-R RRB	-	4.44 *(2.79)*

Includes only participants included in the final analyses

Group differences tested with *T*-tests or Chi-square test

*TD* typical developing, *ASD* autism spectrum disorder, *BRIEF* Behavior Rating Inventory of Executive Function, *MI* Metacognition Index T-Score, *BRI* Behavioral Regulation Index T-Score, *ADOS* Autism Diagnostic Observation Scale Total Score, *SA* social affect, *RRB* restricted, repetitive behavior, *ADI-R* Autism Diagnostic Interview-Revised, *Soc* social, *Comm* communication, *RRB* repetitive and repetitive behaviors, *CSS* Calibrated Severity Score [34]

*Differences where *p* < 0.05

ASD diagnosis was established by expert clinical evaluation (CASD staff) using Diagnostic and Statistical Manual of Mental Disorders Fifth Edition (DSM-5) criteria [1] and confirmed through completion of an Autism Diagnostic Observation Schedule (ADOS; *n* = 9) [46] and/or Autism Diagnostic Interview-Revised (ADI-R; *n* = 11) [47], using the criteria from the NICHD/NIDCD Collaborative Programs for Excellence in Autism [40]. Mean scores are presented in Table 1.

Exclusion criteria were (1) Full-Scale IQ estimate below 80 as measured by the Wechsler Abbreviated Scale of Intelligence (WASI) [82], (2) diagnosis of a neurological disorder or condition (e.g., epilepsy) by parent report, (3) psychiatric diagnoses for TD children based on parental report on the Child and Adolescent Symptom Inventory (CASI) [41], and (4) contraindications for MRI compliance including the presence of metal implants or pregnancy. Additionally, participants with ASD taking stimulant medication (*n* = 3) withheld medication for the 24 h prior to participating and 2 additional ASD participants were taking antidepressant medication which was not withdrawn during participation. All remaining children were not medicated.

#### Behavioral assessments

Parents completed a demographic questionnaire, the CASI for assessing psychopathology exclusion criteria in TD, and the Behavior Rating Inventory of Executive Function (BRIEF) [30]. The BRIEF provides three indices, the Behavioral Regulation Index (BRI) a measure of impairment in regulating emotion, response inhibition, and flexibility; the Metacognition Index (MI) a measure of impairment in working memory, planning and organizing, and actively self-monitoring, and the Global Executive Composite (GEC) a measure of overall executive function utilizing all subscales. Parents also completed the Flexibility Scale [72], a non-normed 50 item questionnaire designed to assess clinically significant levels of behavioral inflexibility in real-world settings, including subscales for routines/rituals, transitions/change, special interests, social flexibility, and generativity as well as a total score as a global measure of flexibility problems. This total raw score from this scale was used for correlating with subsequent brain activation, rather than indices from the BRIEF because it was designed specifically to assess variability in behavioral flexibility in children with ASD. All children were administered the Vocabulary and Matrix Reasoning subtests from the WASI to obtain an estimate of IQ [82].

#### Associative memory task

**Fig. 1:**
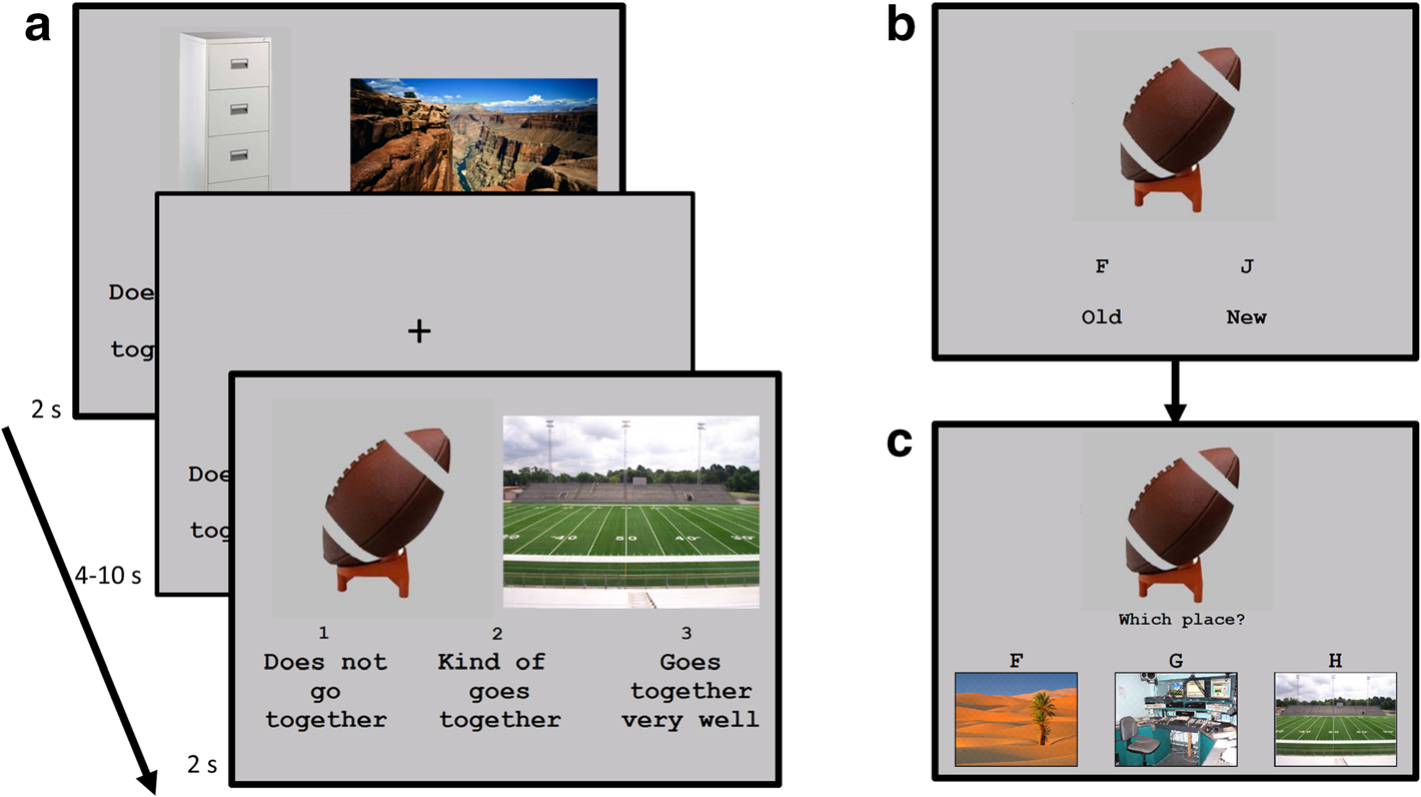
Associative memory task. Encoding task performed by participants. **a** Participants viewed object-scene pairs in the scanner for 2 s with 4–10-s fixations, making a 1–3 discrimination of how well the two go together. After a 20-min delay, participants completed a recognition task outside of the scanner requiring that they **b** first identify if a presented object was new or old and then **c** which scene was paired with the object during the in-scanner encoding

fMRI was performed during the encoding phase of the associative memory task adapted from van Kesteren et al. [75], using 146 of the original 185 object-scene image pairs selected for cultural and age appropriateness (Fig. 1a) which were then randomized into A or B versions of the task. Both versions utilized the same images of objects and of scenes, but the pairings differed such that an object paired with one scene in version A was paired with a different scene in version B, yielding two pairing orders. Following randomization, the A version contained 1 fewer congruent trial than intermediate or incongruent (48, 49, and 49 respectively) while version B contained one fewer incongruent than congruent or intermediate trial based on adult ratings of the pairs. The location of the object on screen was randomized for each task version at creation with the object appearing as the right image during half of the trials and the left image for half.

The stimuli were presented in E-Prime (Psychological Software Tools, Inc., PA, USA) both inside and outside the scanner. Children first completed a practice block on a laptop outside the scanner consisting of 8 image pairs that were not repeated during the in-scanner task. During encoding, participants were presented with object-scene pairs and instructed to choose how well the two images go together from (1) does not go together (representing schema-incongruence), (2) kind of goes together (representing intermediate schema-congruence), to (3) goes together very well (representing schema-congruence) (see Fig. 1a). Three encoding runs were administered, each including 48/49 image pairs and lasting 5:50/6:10 min, respectively, with exposure duration of 2 s and 4–10-s jittered inter-trial intervals. Responses were collected on a button box held in their dominant hand (5 left, 26 right). After a 20-min delay, object recognition (Fig. 1b) and scene association (Fig. 1c) were tested on a laptop outside the scanner, for the 146 encoded pairs in addition to 52 novel pairs. Each trial began with the presentation of an object and required a response of old (the object was seen at encoding) or new (not previously seen). If a participant decided the object was old, they were asked to choose which one of three scenes was associated with the object at encoding (presented alongside it). If the participant decided an object was new, however, participants were not prompted to identify the association, even if the object was old, and instead advanced to the next trial.

### Imaging parameters

Imaging was performed on a 3T Trio Siemens scanner (Erlangen, Germany) at Georgetown University. High resolution T1-weighted structural scans (MPRAGE) were acquired before each of the three trial blocks, lasting 4:18. MPRAGE scans were collected with the parameters of TE/TR = 2.52 ms/1900 ms, TI = 900 ms, 9° flip angle, 1 slab, 1 mm^2^ voxels, 176 sagittal slices with a 1.0-mm thickness, FOV=256 × 256 mm^2^. Each MPRAGE scan was used to align the subsequent functional acquisition to the orientation of the hippocampus to maximize coverage and signal of the MTL. Functional acquisition included a T2*-sensitive gradient echo pulse sequence with the parameters of TE/TR=30 ms/2000 ms, 90° flip angle, 3 mm^2^ voxels, 51 interleaved transverse ascending slices (width = 2.5 mm, gap width = 0.5 mm, effective width = 3 mm).

### fMRI processing

Functional images were analyzed using SPM12 (Wellcome Department of Cognitive Neurology, London, UK) with Matlab 2018a [90]. Preprocessing of the data excluded the first 3 TRs for signal stabilization from each of the three runs, which were subsequently concatenated for further processing. The preprocessing pipeline included slice-time correction, motion correction, co-registration to each participant’s MPRAGE, warping to the MNI template, and smoothing with an 8-mm FWHM Gaussian kernel. Responses were modeled using the temporal derivative and time dispersion alongside the canonical hemodynamic response function. Each function was modeled for six conditions defined at the first level analyses by each of the three levels of congruency separately for subsequently remembered (hits) and forgotten (miss) image pairs yielding 6 condition regressors (congruent hit, congruent miss, intermediate hit, intermediate miss, incongruent hit, and incongruent miss) as well as 10 regressors of no interest: 6 regressors for head motion, 1 to flag and de-weight volumes where total scan-to-scan head motion exceeded 1.5 mm (half voxel) and 3 regressors for run number to control for the concatenation. Level of congruency was classified on an individual basis, using the participant’s ratings at encoding. “Hits” were defined as trials for which the participant accurately identified both the object and its associated scene at recognition, whereas “miss” items were those where either the object or associated scene were not recognized. Contrasts of no interest included hit > miss for each of the three levels of congruency to allow for comparisons of successful encoding across congruency, yielding 3 contrasts (e.g., congruent hit > congruent miss, intermediate hit > intermediate miss, and incongruent hit > incongruent miss). Two participants (1 TD and 1 ASD) were excluded from analyses for lacking any misses in one or more congruency conditions which precluded the calculation of the appropriate hit > miss contrast. One additional ASD participant was excluded for exceeding motion criteria of > 15% of volumes exceeding a 1/2-voxel (1.5 mm) scan-to-scan head movement. The final samples did not differ in either average framewise displacement (ASD = 0.30 mm; SD = 0.15 TD = 0.28 mm; SD = 0.22, *p* = 0.371, BF = 0.35) or in the average number of > 1.5 mm volumes (out of 152 total, ASD = 9.08; SD = 12.43, TD = 14.58; SD = 21.02, *p* = 0.210, BF = 0.39) with Bayes Factors between 1.0 and 0.33 suggesting mild support for null findings and less than 0.33 indicative of strong support for null findings [20].

## Results

### Behavior

#### Demographic and executive function assessment

TD children and those with ASD did not differ on gender composition, age, IQ, or years of maternal education, used as a proxy for socioeconomic status, *p*_s_ > 0.178 (Table 1). Parents rated children with ASD significantly higher than TD children on BRIEF composite index scores, the BRI *t*(30) = 2.19, *d* = 0.838, *p* = 0.043, and the MI *t*(30) = 4.487, *d* = 1.663, *p* < 0.001, GEC *t*(30) = 4.467, *d* = 1.621, *p* < 0.001. Similarly, the ASD group was significantly higher on the Flexibility Scale total raw score relative to TD children (*t*(30) = 4.173, *d* = 1.663, *p* = 0.001), indicating higher difficulty with flexible behavior; this scale is not normed.

#### Associative memory encoding performance

Three measures of encoding task performance were analyzed using R [61]: congruency rating, or the percent of object-scene pairs subjectively rated as congruent (“Goes together very well”), intermediate (“Kind of goes together”), and incongruent (“Does not go together”), rating response time, and an item analysis of congruency rating across participants for intra- and intergroup rating consistency.

**Fig. 2:**
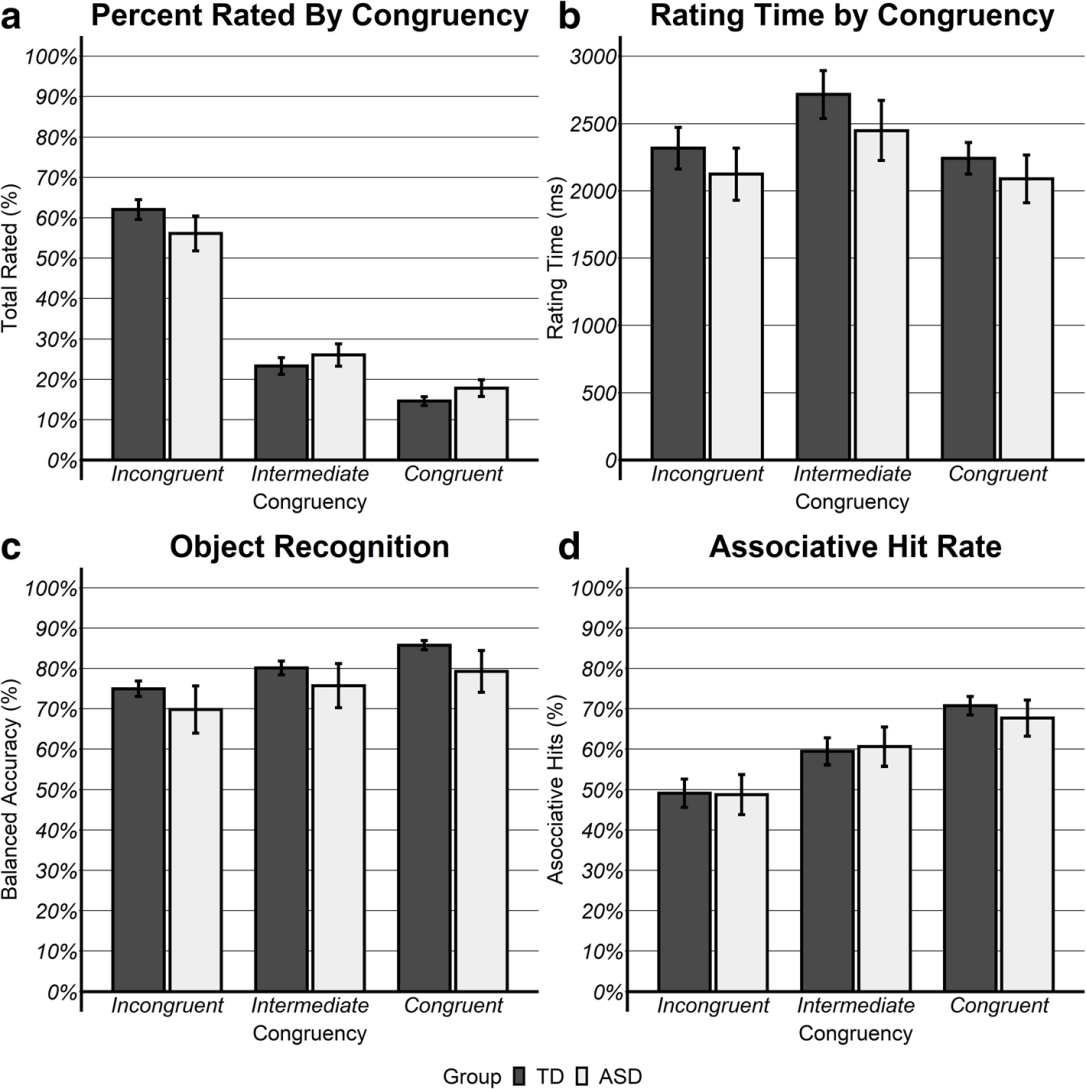
Behavioral performance. **a** At encoding, participants rated significantly more images as incongruent than intermediate or congruent, and more intermediate than congruent. There was no difference, however, between the two groups or interaction with their ratings. **b** Response time for intermediate pairs was significantly slower than incongruent or congruent, without any group differences. **c** There were no significant differences in object recognition due to either congruency or group. **d** The participants remembered significantly more congruent scenes than intermediate or incongruent, and more intermediate than incongruent

First, group differences in the percent of stimulus pairs rated at each congruency level were assessed with a group (ASD, TD) × congruency (congruent, intermediate, incongruent) mixed analysis of variance (ANOVA) with group as a between subjects factor and congruency as a within subjects factor. There was a significant main effect of congruency, *F*(2,57) = 12.80, *η*_p_^2^ = 0.31, *p* < 0.001, such that more pairs were rated as incongruent than intermediate *t*(30) = 9.24, *p* < 0.001, or congruent, *t*(30) = 14.00, *p* < 0.001, and more pairs were rated as intermediate than congruent, *t*(30) = 4.33, *p* < 0.001 (Fig. 2a). Importantly, the main effect of group and its interaction with congruency were not significant (*p*_s_ > 0.810), indicating that the proportions of each rating did not differ between ASD and TD children.

Second, group differences in mean rating response time were assessed at encoding with a group × congruency ANOVA as performed above. There was a main effect of congruency, *F*(2,57) = 3.39, *η*_p_^2^ = 0.11, *p* = 0.04, with significantly slower response times for intermediate pairs than either congruent, *t*(30) = 8.13, *p* < 0.001, or incongruent, *t*(30) = 7.24, *p* < 0.001 — which were not significantly different from each other, *p* > 0.090 (Fig. 2b). Neither the main effect of group nor its interaction with congruency were significant, indicating that response latency for the three levels of congruency did not differ between ASD and TD children.

Third, we assessed agreement of stimuli pair ratings across children both within the TD and ASD groups as well as between them. Spearman rank-order correlations were computed between each participant’s ratings (1, 2, or 3 corresponding to incongruent, intermediate, and congruent, respectively) across the 146 stimulus pairs and the ratings of every other participant (see Supporting Information (SI) Figure 1 for correlation matrix). Next, *t*-tests compared the mean rho values between and across groups — the within-group mean correlation for the ASD group (mean *ρ* = 0.55, SD = 0.15) did not differ from that for the TD group (mean *ρ* = 0.58, SD = 0.11), *p* = 0.376, BF =0.323, indicating that the level of agreement of congruency rating was similar within both TD and ASD groups. Further, the mean within-group correlation (ASD-ASD and TD-TD, mean *ρ* = 0.56, SD = 0.15) did not differ from the mean correlation across groups (ASD-TD, mean *ρ* = 0.52, SD = 0.12), *p* = 0.191, BF = 0.61, further indicating that items were rated similarly by participants. The 0.52–0.58 range of rho values, however, indicates moderate agreement among TD children as well as among children with ASD, suggesting relatively weak schematic knowledge of real-world objects and their context, regardless of diagnostic status, relative to the typical adults the task has been used with in the past.

In sum, performance on the encoding task did not differ between ASD and TD groups in the distribution of stimulus pairs by congruency, rating latency, and agreement in subjective rating across stimulus pairs. Overall, despite our selecting stimulus pairs balanced for adult ratings of congruency, children in both groups rated more stimulus pairs as incongruent than congruent. Ratings of intermediate pairs were in-between, and evoked the longest rating latencies. Further, agreement among children in each group in the level of schema-congruency of stimulus pairs was moderate.

#### Recognition memory performance

Out of scanner memory performance included recognition of objects encoded during scanning and their associated scene. First, for object recognition, percent balanced accuracy for each level of congruency was calculated by averaging the percent object hits and percent correct rejections for lure objects. Balanced accuracy was utilized to correct for the significant imbalance in congruency rating distribution, with over 50% of stimulus pairs rated as incongruent rather than intermediate or congruent. Second, for correctly recognized old objects, the percent of correctly recognized scene associations was calculated as association hits.

A group (ASD, TD) × congruency (congruent, intermediate, incongruent) ANOVA for percent balanced accuracy yielded a significant main effect for congruency *F*(2,57) = 4.84, *η*_p_^2^ = 0.15, *p* = 0.010, such that children exhibited better recognition accuracy for objects in congruent than in incongruent *t*(59) = 3.09, *p* = 0.003 pairs, with no difference relative to intermediate, *p*s > 0.231. The main effect of group reached trend level significance, *F*(2,57) = 3.72, *η*_p_^2^ = 0.12, *p* = 0.057, such that TD children exhibited better object recognition than ASD children (Fig. 2c). The group × congruency interaction did not reach significance, *p* = 0.700.

A group × congruency ANOVA for association hits revealed a significant main effect for congruency, *F*(2,87) = 29.69, *η*_p_^2^ = 0.405, *p* < 0.001, with children exhibiting better recognition for congruent scenes than intermediate *t*(30) = 2.55, *p* = 0.013, and incongruent *t*(30) = 6.73, *p* < 0.001, as well as better recognition for intermediate than incongruent *t*(30) = 4.93, *p* < 0.001. Further, there was neither a significant main effect for group nor an interaction with congruency (*p*_s_ > 0.12), indicating that the percent of overall association recognition and differences by congruency were similar between groups (Fig. 2d).

In sum, as predicted, TD children exhibited enhanced memory for both objects and associated scenes that were congruent with schemas than incongruent. Contrary to prediction of deficits, ASD children also demonstrated schema-sensitivity memory facilitation, although a marginally significant group difference suggested lower object memory in children with ASD.

### Imaging

The analytic approach for the imaging data was delineated into two stages. First, voxel-wise analyses for schema-sensitive activations associated with subsequently remembered object-scene pairs were performed in anatomical masks for the mPFC and MTL for testing hypotheses and in the whole brain for exploratory purposes. Second, voxel-wise analyses were performed assessing the interaction of Flexibility Scale raw scores with congruency to test the hypothesis about the relationship between behavioral flexibility and schema-based memory formation in mPFC and subsequently in an exploratory whole-brain analysis.

#### Schema evoked

Three anatomical masks were defined using the AAL atlas, the left and right MTL which included the hippocampus and parahippocampus, and the mPFC which encompassed bilateral anterior cingulate gyrus, gyrus rectus, and frontal medial orbital gyrus. For each mask as well as in the whole brain, one-way ANOVAs were performed to identify voxels differing by congruency (congruent, intermediate, incongruent) for the associative hits > miss contrast, separately for each group with GLM Flex Fast2 (http://mrtools.mgh.harvard.edu/). To control for multiple comparisons, 3dclustersim was used for each mask to determine significant cluster size at *p* < 0.05 for the MTL (2-sided, nearest neighbor 2; *p* = 0.001; *k* = 9) and the mPFC (2-sided, nearest neighbor 2; *p* = 0.005; *k* = 47) masks, and for the whole brain (2-sided, nearest neighbor 2; *p* = 0.005; *k* = 159). Groups were directly compared with group × congruency ANOVAs in each mask and the whole brain.

**Fig. 3:**
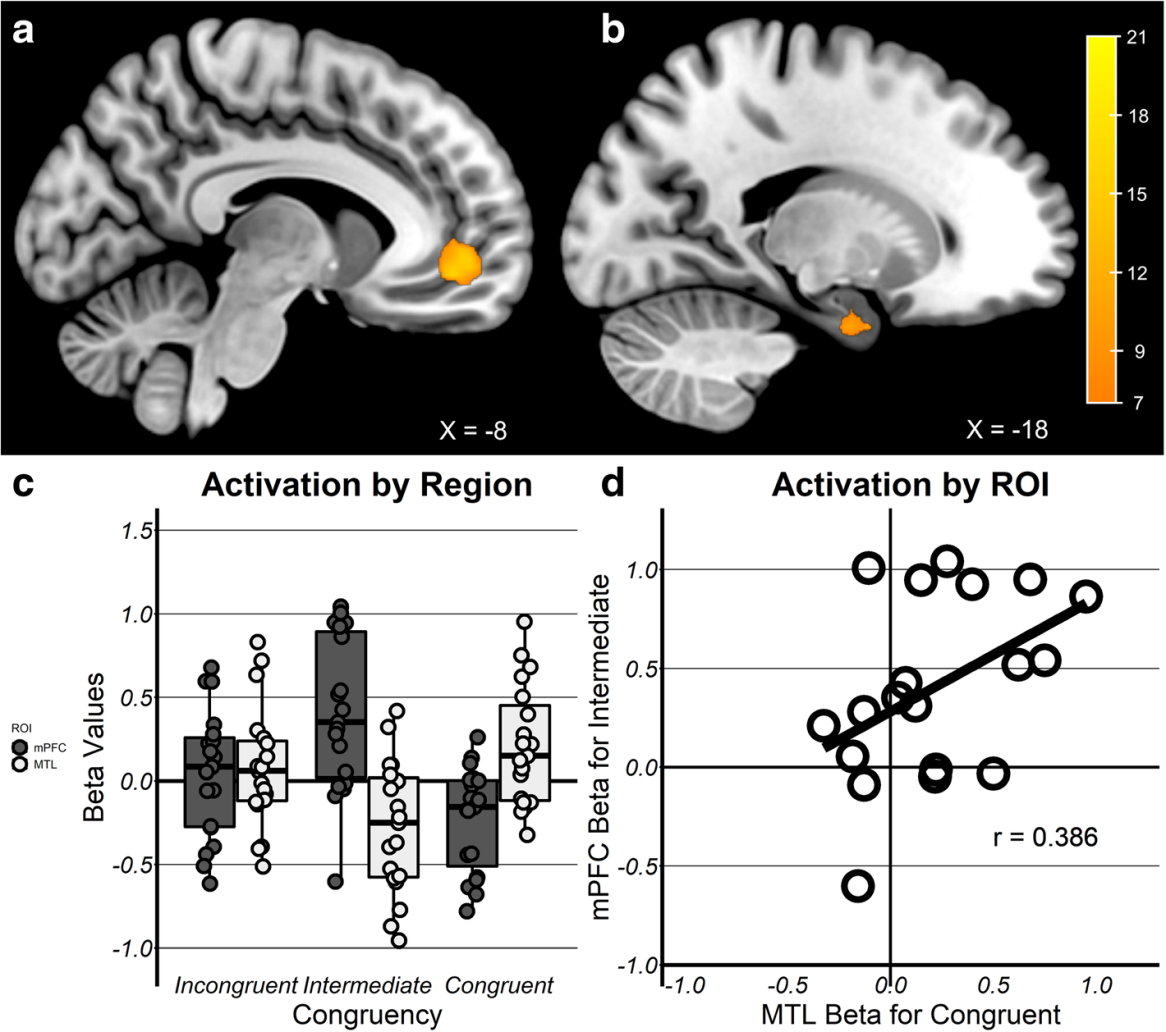
Schema-dependent activation in TD children. **a** TD children exhibited a cluster sensitive to schema-congruency in the mPFC and **b** left MTL (lMTL). **c** Congruency × ROI interaction indicating a trade-off by congruency such that memory for intermediate pairs was supported by mPFC (dark bar) and congruent pairs by lMTL. **d** Trade-off showing lMTL activations in Congruent trials correlated with mPFC activations for Intermediate trials indicating the degree of mPFC engagement is related to left lMTL engagement

First, results in TD children showed a significant cluster sensitive to congruency (Fig. 3a) in the mPFC, specifically within the pregenual anterior cingulate (*k* = 207, peak = − 3, 48, 0) spanning BA10/11/32. The left MTL also exhibited a significant cluster (Fig. 3b) sensitive to congruency within the anterior hippocampus (*k* = 10, peak = − 15, − 6, − 27) spanning both the hippocampus and parahippocampus. No significant clusters were observed within the right MTL mask. These results were also observed when the ANOVAs were repeated to control for age, gender, and performance differences likely to influence activation, such as number of remembered pairs and reaction time at encoding (see SI Figures 2, 4, 6, 8).

Second, in order to determine whether mPFC and MTL traded off by congruency condition, they were directly compared by conducting a region of interest (ROI: mPFC, left MTL) × congruency (congruent, intermediate, incongruent) repeated measures ANOVA for the contrast estimates in each ROI. Estimates were extracted from the two masks defined by the observed clusters from the TD group analyses. Mean signal was extracted from each participant, for each level of congruency within the observed group-level cluster. While main effects were not significant (*p*s > 0.406), a ROI × congruency interaction *F*(2, 57) = 15.9, *η*_p_^2^ = 0.36, *p* <0.001 was observed (Fig. 3c), such that mPFC activation was higher during encoding of intermediate pairs relative to congruent pairs *t*(30) = 4.30, *p* < 0.001, whereas left MTL activation showed the reverse, higher during encoding of congruent pairs relative to intermediate pairs *t*(30) = 3.37, *p* = 0.001; incongruent pairs did not differ significantly from congruent pairs for either region (*p* = 0.254). At each level of congruency, the two regions differed significantly for congruent pairs (left MTL > mPFC), *t*(30) = 3.17, *p* = 0.002, and for intermediate pairs (mPFC > left MTL), *t*(30) = 4.87, *p* < 0.001, but not for incongruent pairs (*p* = 0.117). Additionally, we determined whether the observed trade-off by congruency between the two regions was present at the individual level. A significant correlation was found between activation in the mPFC for intermediate pairs and the MTL for congruent pairs (*r* = 0.386, *p* =0.003) (Fig. 3d).

Third, in the ASD group, no voxels sensitive to congruency reached the corrected level of significance in either MTL mask or the mPFC. Similarly, direct comparison of the TD and ASD groups with a group by congruency ANOVA within each of the three masks yielded no significant clusters that survived correction.

Fourth, whole-brain analysis revealed that only one region differed by congruency in the TD group, the same mPFC cluster (*k* = 207, peak = − 3, 48, 0) that was observed with the mask-limited analysis. Similar to the mask-limited analysis, in children with ASD, no significant clusters sensitive to congruency were found to survive whole-brain correction. Finally, the two-way ANOVA yielded no significant interaction or main effects for either group or congruency.

In sum, TD children exhibited mPFC and MTL engagement as a function of congruency, preferentially engaging the anterior left MTL during encoding of subsequently remembered congruent pairs and the mPFC for intermediate pairs, with no difference for incongruent pairs. Further, the mPFC cluster survived whole-brain correction as well. In contrast, children with ASD did not exhibit any significant activation sensitive to schema-congruency during encoding of subsequently remembered object-scene pairs in either mPFC, MTL, or the whole brain.

#### Association with flexibility

Two analyses were conducted to examine the association between schema-based memory encoding and behavioral flexibility. An absence of significant differences based on congruency in the ASD children in the mPFC and MTL could be a result of high individual variability in activation. We tested whether the Flexibility Score accounted for individual differences in activation in those regions. First, in order to test the hypothesis that mPFC engagement is associated with behavioral flexibility in children with ASD, a voxel-wise congruency (congruent, intermediate, incongruent) × flexibility (Total Score continuous variable) ANOVA was performed with GLMFlex Fast 2, separately in the mPFC and MTL mask. Secondly, this same ANOVA was also performed in the whole brain to explore additional regions supporting congruency-sensitive encoding that vary by flexibility for the children with ASD. Due to the design of the Flexibility Scale, TD children exhibited a pronounced floor effect that precluded enough variability to probe a relationship between activation and behavioral flexibility.

**Fig. 4:**
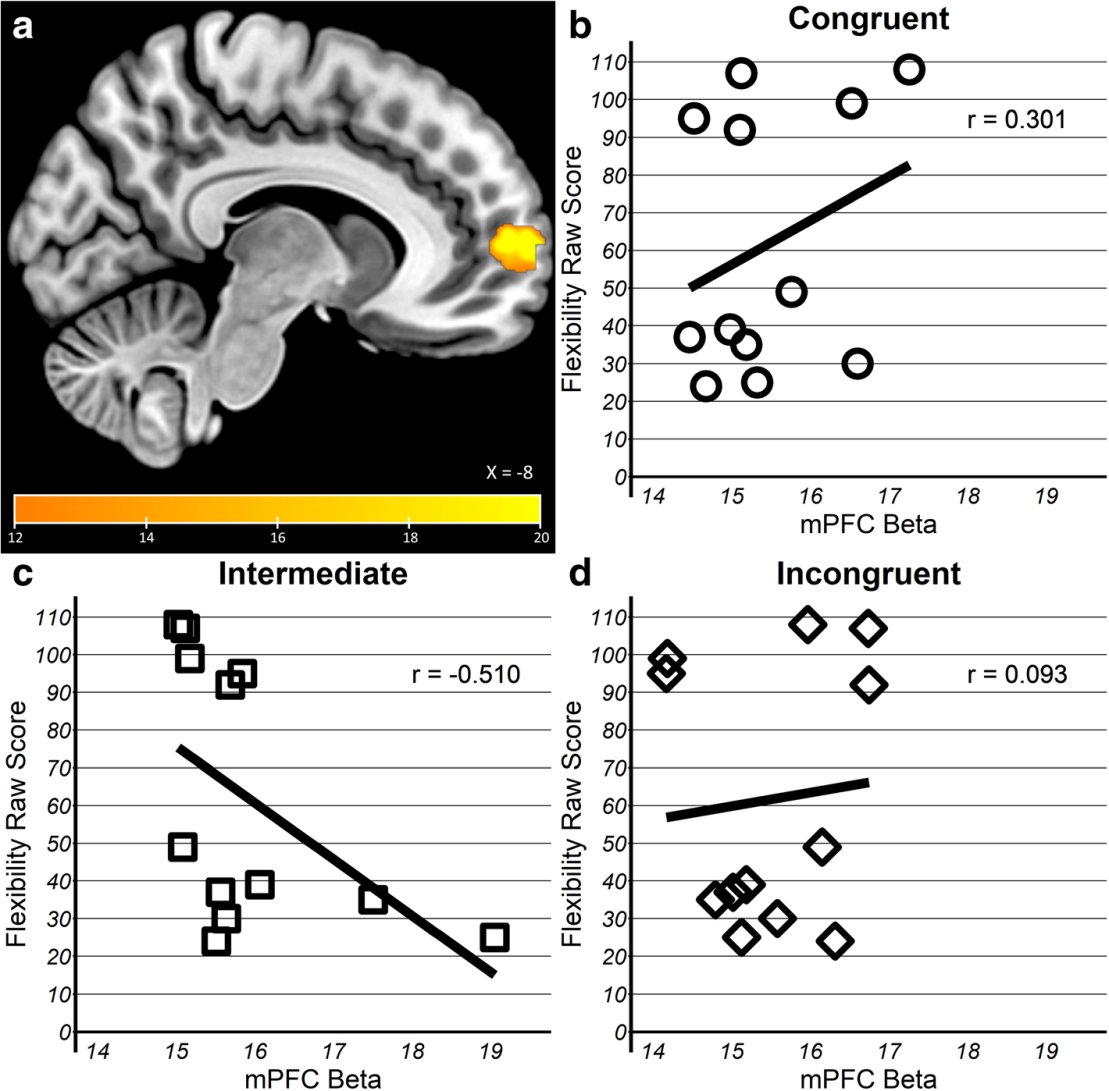
Schema-dependent activation in ASD varied by behavioral flexibility. **a** Congruency × flexibility interaction was observed in a cluster in mPFC. Extracted beta values from mPFC cluster showing **b** a mild positive relationship between inflexibility (higher score) and activation congruent, **c** a moderate negative relationship for Intermediate, and **d** minimal relationship for incongruent trials

First, the mPFC mask-based analysis revealed a congruency × flexibility interaction in one cluster (*k* = 49, peak =9, 63, 6) anterior to the cluster observed in one-way ANOVA for congruency in TD children (Fig. 4a), while there were no clusters that reached corrected threshold within the MTL masks. Within the mPFC cluster, association with individual differences in behavioral flexibility varied such that activation during encoding of successfully remembered object-scene pairs of intermediate congruency was moderately negative (*r* = − 0.51) whereas it was weakly positive for congruent (*r* = 0.30) and incongruent (*r* = 0.09) pairs (Fig. 4b-d). Therefore, children with ASD who were more flexible in everyday behavior (lower Flexibility Total Score) engaged the mPFC more for intermediate pairs, similar to the TD children. These results were also observed when the analyses were repeated to control for age, gender, and performance differences likely to influence activation, such as number of remembered pairs and reaction time at encoding (see SI Figures 3, 5, 7, 9).

**Fig. 5:**
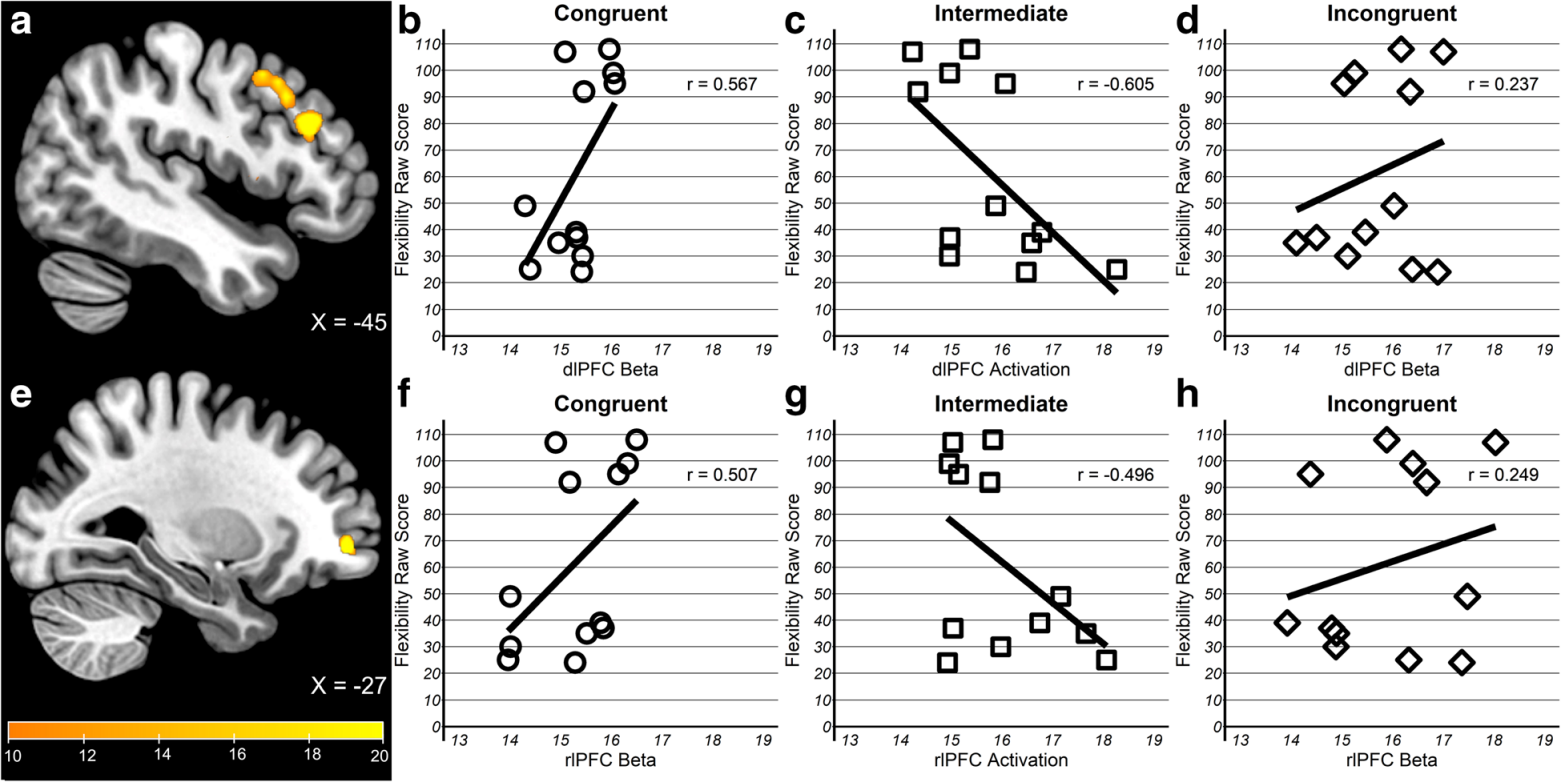
Flexibility-dependent schema activation in left dlPFC and rlPFC for children with ASD. Results from whole-brain, exploratory analysis yielding **a** dlPFC cluster with **b**–**d** flexibility exhibiting minimal to moderate positive relationships between flexibility score and activation in congruent and incongruent pairs by a moderate negative relationship in Intermediate pairs and **e** rlPFC cluster with **f**–**h** flexibility exhibiting minimal to moderate positive relationships between flexibility scorer and activation in congruent and incongruent pairs by a moderate negative relationship in intermediate pairs

Second, in whole-brain analysis, no clusters showed a congruency × flexibility interaction at the corrected threshold (*p* < 0.005, *k* = 159); however, two subthreshold clusters were observed in left dorsolateral prefrontal cortex (dlPFC, *k* = 96, peak = − 45, 27, 24) spanning BA8/9 and an anterior cluster in left rostrolateral prefrontal cortex (rlPFC, *k* = 23, peak = − 27, 51, − 3) spanning BA10/46 (Fig. 5). Similar to the mPFC cluster reported above, activation during encoding intermediate pairs in both regions showed a moderate negative correlation with flexibility (dlPFC, *r* = − 0.61; rlPFC, *r* = − 0.50), whereas it was moderately positive for congruent (dlPFC, *r* = 0.56; rlPFC, *r* = 0.51) and weakly positive for incongruent (dlPFC, *r* = 0.24; rlPFC, *r* = 0.25) pairs.

In sum, schema-based encoding of subsequently remembered information was associated with behavioral flexibility in ASD. Children with ASD engaged mPFC during encoding of object-scene pairs of intermediate congruency similar to TD children; however, the extent of engagement was higher in those who exhibited more flexible behavior in everyday life. Further, two lateral frontal regions, dlPFC and rlPFC, which were not observed in typical schema encoding in TD children, also exhibited a similar association with flexibility, albeit, less reliably.

## Discussion

Our preliminary investigation revealed four main findings regarding the neural correlates of schema-based memory formation in late childhood and adolescence and in relation to behavioral flexibility. First, as hypothesized from adult reports, schema-congruency facilitated associative memory in TD children and distinguished encoding-related activation for subsequently remembered associations in mPFC and left anterior MTL. In contrast to adult reports, however, mPFC was engaged during encoding of object-scene pairs rated as intermediate relative to those rated as congruent, whereas left MTL was engaged during encoding pairs rated as congruent relative to those rated as intermediate. Second, in contrast to the hypothesis, associative memory performance in children with ASD was facilitated by schema-congruency similar to TD children; however, they had overall lower object memory. Third, as hypothesized, children with ASD who were rated higher (better) by parents on behavioral flexibility engaged mPFC in a manner consistent with TD children — greater recruitment during the encoding of subsequently remembered intermediate pairs. Fourth, two lateral prefrontal regions, not observed in TD children or previously with TD adults, also showed an association with flexibility in children with ASD in exploratory whole-brain analysis. We recognize that these results are based on a small sample and therefore, consider them highly preliminary. Their value, however, is in suggesting the viability of a conceptual framework unifying the formation of abstract mnemonic representation and behavioral adaptation to novel settings. While this link has been made theoretically, the present findings lend empirical support for opening up larger investigations for exploring mechanisms by which abstract representation may promote flexible behavior.

Our behavioral results shed new light on schema-related associative memory in children. A unique strength of our design was the examination of schema-congruency effects based on subjective ratings, which accommodate idiosyncratic notions of object-scene associations. Rating agreement among children in each group was moderate, indicating heterogeneity in schemas of real-world objects. Such heterogeneity may be representative of incomplete or weak schema formation during a period when schemas are actively under construction with increasing number of life experiences. If this reflects developmental immaturity, rating agreement ought to increase with age. This prediction was borne out in the TD sample (*r* = 0.309, *p* < 0.001), but not in the ASD sample (*r* = 0.060, *p* = 0.607), perhaps due to sample size constraints or atypical developmental trajectories of schema formation in ASD. One disadvantage of subjective congruency classification is that the proportion of pairs classified at each congruency level could not be controlled. While the included stimuli-pairs were balanced across congruency levels based on adult norms, autistic and TD children classified many more pairs as incongruent relative to intermediate or congruent. While this imbalance did not influence memory measurement because we calculated balanced object recognition accuracy, the departure from an adult-like distribution by congruency suggests immature schematic knowledge, perhaps also resulting from limited real-world experience compared to adults.

Object and association recognition were facilitated by schema-congruency as expected in TD children, and unexpectedly, also in autistic children. Previous findings show associative memory facilitation by higher congruency with schematic knowledge in both TD children [9, 21, 54] and adults [55, 78]. The facilitation observed in ASD children was unexpected based on prior literature showing diminished or absent schema facilitation in memory performance [11, 48]. However, these prior studies rely largely on free recall or word recognition and often have encoding-recall delays of an hour or more. This is important given that differences in task structure can manipulate the strength of congruency-based memory facilitation, including our use of recognition rather than free recall and changes in the proportion of trials creating an “isolation effect” whereby there is better memory for the lowest-proportion category [16, 78]. As fewer pairs were rated as congruent than incongruent, these proportion differences may have boosted memory for congruent pairs on our pictorial recognition task. As a result, the design may be particularly strong in drawing out schema-based memory facilitation even in a group where it is not typically observed. Nevertheless, facilitation of object recognition and associative memory in both autistic and TD children suggests sufficient schema development to support memory formation.

Despite behavioral similarities with adult reports, our imaging findings revealed that mPFC and MTL was recruited by different congruency conditions than adults. Specifically, Van Kesteren et al. [75] observed a trade-off between mPFC and MTL by congruency, with preferential mPFC engagement for congruent and MTL for incongruent pairs. Our TD children showed a different pattern of trade-off, with preferential mPFC engagement for intermediate and MTL for congruent pairs (Fig. 3). Additional analyses verified that the results were not an artifact of age or gender distribution or differences in either encoding rating latency or number of associative hits trials (see SI). Neural correlates in autistic children were similar to TD children in that mPFC was engaged during encoding of subsequently remembered pairs of intermediate congruency, but it depended on degree of behavioral flexibility — children rated as more flexible by parents had higher mPFC activation. As with the TD findings, these results held after controlling for age, gender, response time, and associative hit trials (see SI). Association with behavioral flexibility was not observed for the MTL. Additionally, more flexible children with ASD also recruited the dlPFC and rlPFC for intermediate pairs, a region not observed in TD children or implicated in previous studies with adults. The low scores and small range in TD children precluded the ability to test whether the same effect would be observed in TD children. In drawing comparison with Van Kesteren et al. [75] study, it is important to note that we used the same stimuli but modified the design for suitability to our younger participants. We reduced the encoding-recognition delay from 1 day to 20 min, used pictures as recognition probes rather than single-word descriptions (i.e., representing of the image of a playroom rather than just presenting the word ‘Playroom’), and used a three-level congruency scale rather than a continuous rating scale. It is unclear whether these design differences led to the observed distinct pattern of neural recruitment. A parsimonious interpretation, in light of the adult-like pattern of memory performance, is that the observed mPFC and MTL trade-off between intermediate and high levels of congruency represents a developmental difference, with findings from ASD providing the first evidence for linkage between schema encoding and behavioral flexibility.

Two theoretical frameworks that converge on the relationship between mPFC and MTL in representing abstract knowledge are useful for considering the present findings. The Schema Linked Interactions between Medial prefrontal and Medial temporal regions (SLIMM) framework proposes that reciprocal communication between the mPFC, which represents associative frameworks of knowledge or schemas, and MTL, which represents non-associative information, underlies the formation and use of schema knowledge [78]. As schemas are formed and strengthened through reactivation, the mPFC detects schema-congruent information and inhibits engagement of the MTL. It is hypothesized that the inhibition of the MTL prevents interference that can arise from reactivation of numerous past, similar representations [68, 78]. Category learning represents a second model that provides a complementary lens to interpret our findings as category knowledge has been posited to be a simple form of schema [29, 57, 63]. Categories are formed by extracting prototypical information, with generalized representations of a category encoded by the mPFC and exemplar-specific information encoded by other regions, including the MTL (for review see [87]). Both schema and prototype knowledge are representations which are extracted from elements across experiences and are designed to be flexibly utilized in the accommodation of new information [27, 67, 88].

Our findings suggest a downward extension of the above frameworks to accommodate findings from children that reflect weaker schema representations. Stimulus pairs of intermediate congruency had longer classification response times suggesting effortful evaluation. mPFC is known to play a role in deliberative cognition in the face of uncertainty [65]. Its engagement for the intermediate (rather than congruent) pairs in children may reflect the deliberative evaluation necessary in the face of weakly instantiated schemas. This finding is consistent with the hypothesized role of the mPFC as a detector for schema-congruency. Less clear, however, is the seemingly opposite pattern of MTL engagement for subsequently remembered congruent pairs in children relative to incongruent pairs in adult studies. It is important to note that the observed MTL cluster lies anterior to that reported in adults by Van Kesteren et al. [75], with no overlap. The anterior MTL is associated with gist representations in adults [17, 29], which are context-associative representations that provide the basis for schema development [68]. Thus, developing brains may lack sufficient inhibition from the mPFC to MTL, and as predicted by SLIMM, the observed anterior MTL response likely represents reactivated gist representations that are similar to the congruent associations. In sum, anterior MTL and mPFC engagement during memory formation of object-scene associations of high and intermediate congruency in children, represent an immature state of schema representation.

It is important to note that our findings stand in contrast to the only other study to have examined mPFC engagement during schema-dependent encoding in TD children using a similar object-scene association paradigm [15]. Similar to our findings, they reported behavioral schema-based memory facilitation for congruent pairs in both the adults and children following an incidental encoding task where participants were asked to decide if object-scene pairs were congruent or incongruent. Unlike our findings, they reported greater mPFC activation for subsequently remembered congruent pairs, without an effect of age. Notable differences in the design of their study relative to ours are inclusion of binary encoding classification (congruent or incongruent only), and younger (6–7 years old) and older (18–22 and 67–74 years old) participants than in our study. In light of our finding of moderate encoding ratings agreement, it is likely that the younger sample in Brod and Shing’s study rated as congruent those pairs which would have been rated as intermediate were that option available. In such a case, the activation associated with the deliberative evaluation may be captured in their congruent trials. It is not clear though, if children are more likely to treat cases of intermediate schema-congruency as congruent or incongruent. As a result, our findings represent a greater spectrum of congruency that may be obscured in a binary approach of congruent or incongruent ratings if there is a bias towards considering moderate congruency as congruent rather than incongruent. Nonetheless, taken together these studies highlight the need for examining a broad spectrum of schema-congruency with dimensional rather than categorical classification.

While our sample size is underpowered, we consider the main contribution of our findings to suggest a novel line of investigation in ASD, the relationship between abstract mnemonic representation and executive function. mPFC activation during encoding of subsequently remembered object-scene pairs was observed in the subset of autistic children who exhibited more flexibility in everyday behavior. These neural correlates were typical, as mPFC was more engaged for intermediate congruency, just as in their TD peers. While this dependence on behavioral flexibility supports the association between abstract knowledge representation and flexible adaptation to the environment posited by the theoretical models [27, 64, 66], its raises the question of causality — does good executive function promote typical abstract representations or does the ability to represent abstract knowledge typically by mPFC promote better adjustment to real-world challenges? This question cannot be adjudicated by the present results and requires longitudinal designs to separate cause and effect. Furthermore, our results also reveal the potential contribution of lateral prefrontal cortex in flexibility-dependent schema-based memory formation. In exploratory analysis, left rlPFC and dlPfC were recruited by the flexible children to a greater extent along with mPFC. While mPFC has been implicated in associative, prototype-based learning, both the dlPFC and rlPFC have been implicated in rule-based, exemplar learning [87]. The rlPFC is preferentially engaged in category learning tasks when individuals are able to learn a finite number of classification rules [56] and has also been implicated in flexible cognition such as multitasking [28], integration of relational association and analogical reasoning [18, 84], and episodic memory retrieval [19, 71]. Similarly, the dlPFC has also been implicated in rule-based learning tasks [5] and task switching [35]. While it is important to consider this association preliminary given the sample, these results suggest a testable hypothesis for future work — that more flexible autistic children utilize two parallel representations in tandem, abstract and rule-based, for memory formation of ambiguous associative information.

Our results in ASD conform to the broader literature of structural and functional differences in both the MTL and mPFC and provide some clinical implications. Both primary ROIs for this study are components of the Default Mode Network (DMN). The DMN is a functional network important for theory of mind, introspection, and prospective planning [2, 62]^,^ which are known deficits in ASD [4, 22, 43]. Autistic children consistently show mPFC underconnectivity relative to TD [3, 36, 50, 81, 85]. Given the link between both schema-dependent activations in and functional connectivity strength between the MTL-mPFC [76, 77], our findings suggest a novel lens to view previous DMN findings, especially in light of the association between mPFC-DMN underconnectivity and cognitive flexibility in set-shifting tasks [79]. Thus, known DMN disruptions in ASD all converge on the mPFC. While less is known about the functional connectivity of the MTL in ASD, greater connectivity between the MTL and posterior cingulate was associated with greater symptom severity [52] and greater hippocampal volume has been observed throughout childhood in ASD [6, 31, 80].The relationship between schema use, flexibility problems, and DMN disruptions, however, remains to be characterized in ASD. Our preliminary results begin to offer an interpretation of real-world flexibility problems observed in some children with ASD. The apparent bimodal distribution of flexibility scores of our ASD aligns with findings revealed by data-driven methods suggesting subtypes distinguished by flexibility problems [74]. The bimodal distribution also points to limitations of instruments to measure the full spectrum of behavioral flexibility. Problems with flexibility in behavior and associated abstract knowledge representation may characterize a subset of ASD children and moreover may not be limited to ASD but broadly represented across disorders with executive dysfunction.

Conceptually, further investigation of linkage between abstract mnemonic representation and executive function has the potential to inform clinical practice. By demonstrating that learning in children with ASD *can* be facilitated by schema-congruency under specific conditions (use of concrete pictures for recognition and allowance of subjective determination of which items are congruent), this study highlights the importance a strengths-based approach that probes the conditions under which a specific type of learning *can* occur. This is essential if the long-term goal is to improve learning and memory in children with developmental disabilities. The identification of flexibility as a correlate of PFC engagement during learning for children with ASD would lead to hypotheses regarding the potential of flexibility training, an evidence-supported intervention in ASD [37], to normalize brain function during learning.

## Conclusions

The present study is the first to characterize neural representations of schema in children in late childhood and adolescence and the first to provide evidence, albeit preliminary, of differences in schema-dependent brain activations in children with ASD and the connection between behavioral flexibility and schema. The generalization of our findings is limited by the relatively small sample of children with ASD and extrapolation to observed adult findings due to a lack of direct developmental comparison group of adults. Further work is required to fully understand developmental trajectories of schema models and assess flexibility through assessments more sensitive to variability of flexibility in non-clinical samples. Nevertheless, this study provides the first important link.

## Supplementary Information

**Additional file 1: **Supporting Information. Encoding Performance – Rating agreement across participants: **Supporting Information (SI) Figure 1.** Correlation matrices for rating agreements. Imaging – Control analyses: **SI Figure 2.** Congruency differences in TD children after controlling for age. **SI Figure 3.** Congruency differences in ASD children after controlling for age. **SI Figure 4.** Congruency differences in ASD children after controlling for age. **SI Figure 5.** Congruency differences in ASD children after controlling for the number of associative hits. **SI Figure 6.** Congruency differences in TD children after controlling for response time. **SI Figure 7.** Congruency differences in ASD children after controlling for response time. **SI Figure 8.** Congruency differences in TD children after controlling for gender. **SI Figure 9.** Congruency differences in ASD children after controlling for gender.

## Data Availability

The datasets used and/or analyzed during the current study are available from the corresponding author on reasonable request.
