# Supplementary material for: Neural correlates of schema-dependent episodic memory and association with behavioral flexibility in autism spectrum disorders and typical development
Source: J Neurodev Disord. 2021 Sep 15;13:35. doi: 10.1186/s11689-021-09388-9 (PMC8442441; doi:10.1186/s11689-021-09388-9)
Supplement: Supplementary file 1 — Additional file 1: Supporting Information. Encoding Performance – Rating agreement across participants: Supporting Information (SI) Figure 1. Correlation matrices for rating agreements. Imaging – Control analyses: SI Figure 2. Congruency differences in TD children after controlling for age. SI Figure 3. Congruency differences in ASD children after controlling for age. SI Figure 4. Congruency differences in ASD children after controlling for age. SI Figure 5. Congruency differences in ASD children after controlling for the number of associative hits. SI Figure 6. Congruency differences in TD children after controlling for response time. SI Figure 7. Congruency differences in ASD children after controlling for response time. SI Figure 8. Congruency differences in TD children after controlling for gender. SI Figure 9. Congruency differences in ASD children after controlling for gender. [file 11689_2021_9388_MOESM1_ESM.docx]

**Supporting Information**

**Encoding Performance – Rating agreement across participants**


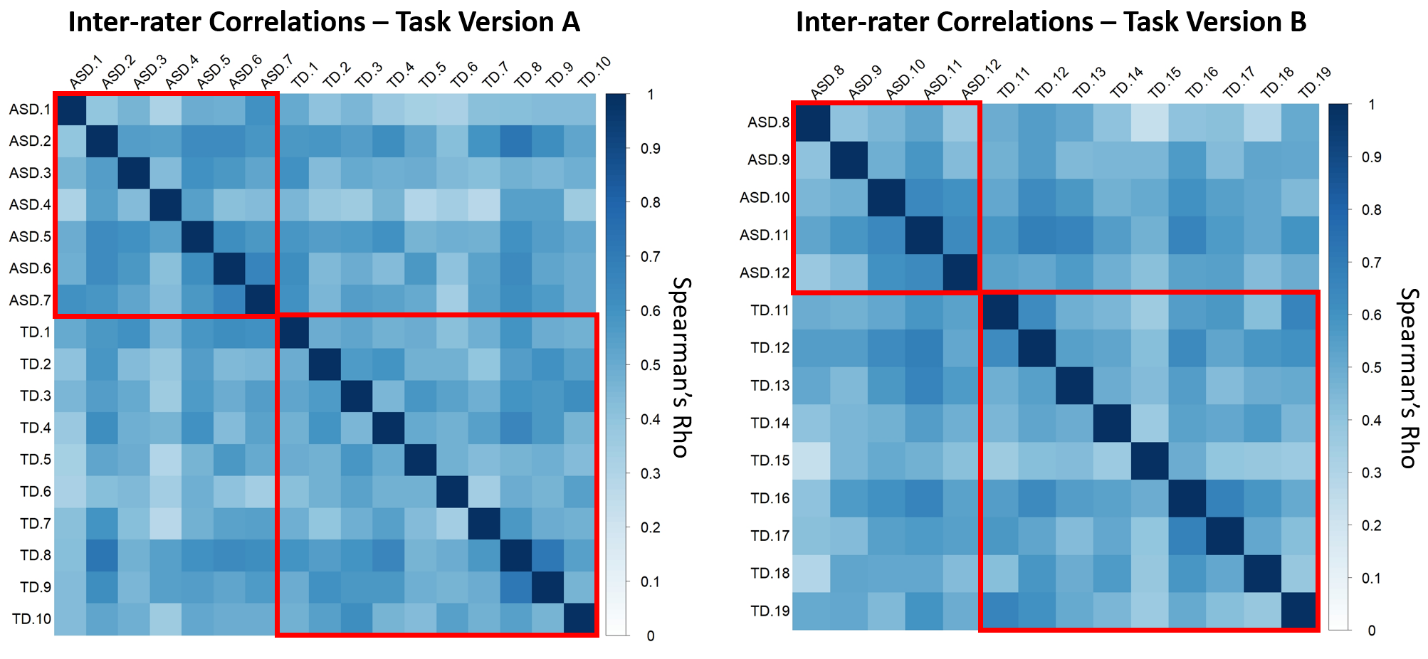
Each participant’s (ASD.1-12 & TD.1-19) ratings of congruency (incongruent-1, intermediate-2, and congruent-3) across the 146 stimuli pairs were correlated with every other participant within the same task version (A,B), yielding a Spearman’s Rho values for each participant with every other participant (SI Figure 1).

*Supporting Information (SI) Figure 1:* *Correlation matrices for rating agreements*

*Spearman rho values are color-coded (0-1) and grouped in the appropriate task version (A,B); no negative correlations were observed. Red squares mark within group (TD-TD and ASD-ASD) correlations while values outside correspond to between group (ASD-TD) correlations.*

Subsequent t-tests were performed on rho values to assess within and between group agreement. Differences for within group agreement was assessed by comparing average ASD-ASD (mean ρ = 0.55, SD = 0.15) to average TD-TD (mean ρ = 0.58, SD = 0.11) rhos, while between group agreement was assessed by comparing the average within group (ASD-ASD and TD-TD combined, mean ρ = 0.56, SD = 0.15) to between group (ASD-TD, mean ρ = 0.52, SD = 0.12) rhos. There were no significant differences between within-group agreement of the TD and ASD participants, *t*(59) = 0.8, p = 0.376, and between the between-group agreement and the within-group agreement, *t*(59) = 0.5, p=0.191. While the moderate rho values (0.52-0.58) indicate significant heterogeneity in ratings between participants, the absence of any significant differences in the t-tests suggest the two groups show consistent levels of agreement within and between groups, and therefore all groups can be said to exhibit no differences in their agreement in rating image pairs.

**Imaging – Control analyses**

In order to assess the potential confounding effects of differences in demographic (age and gender) and performance (classification rating response time at encoding, total number of hits) variables, the ANOVA examining differences by Congruency (Congruent, Intermediate, Incongruent) in TD children and the Congruency X Flexibility ANOVA in children with ASD were repeated with each of the confounding variables. These Analyses of Covariance (ANCOVAs) were performed in the left MTL (p < 0.05 corrected threshold of k = 9 at p < 0.005) and mPFC (p < 0.05 corrected threshold of k = 58 at p < 0.005) masks separately for each group.

First, we wanted to determine whether the observed mPFC and left MTL activations were a function of maturational differences, in light of the relatively wide age range of the samples (8 – 15 years). The ANCOVAs including age replicated the results of the ANOVAs without age in TD, mPFC (k = 207, peak = -3, 48, 0) and MTL (k = 9, peak = -15, -6, -27) (SI Figure 2) and the Congruency X Flexibility interaction in ASD in a same mPFC cluster (k = 44 peak = 9, 63, 6), which exhibited the same relationship between flexibility and the three levels of congruency (SI Figure 3). Thus, these results indicate that the observed results in the main text did not depend on maturational differences.

*
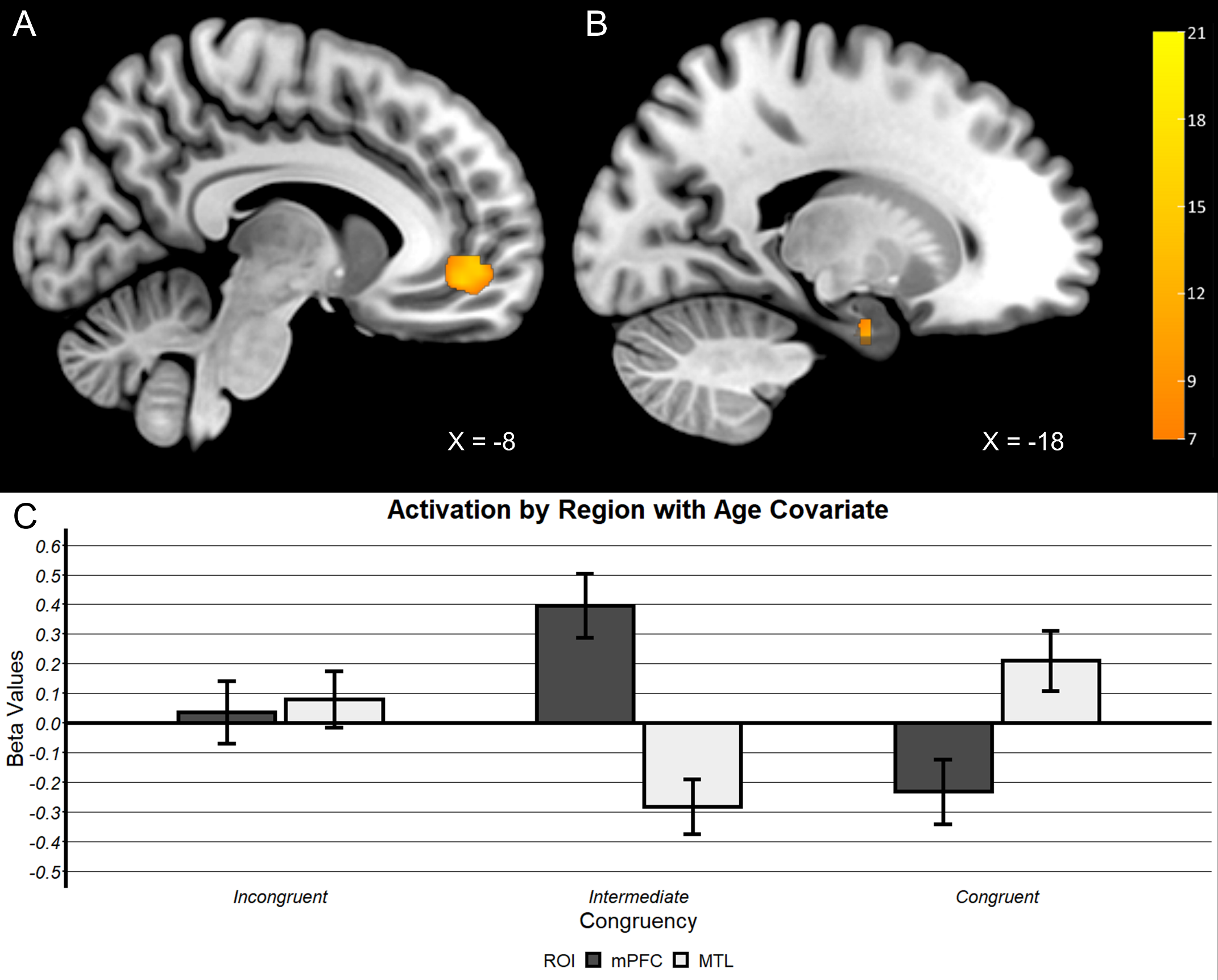
 SI Figure 2: Congruency differences in TD children after controlling for age*

*Results of a one-way ANCOVA by Congruency (Congruent, Intermediate, Incongruent) with age as a covariate in the TD sample with the Associative Hits>Miss trials contrast, in the mPFC mask (A) and the left MTL mask (B); extracted beta values from each cluster are plotted in (C).*

*
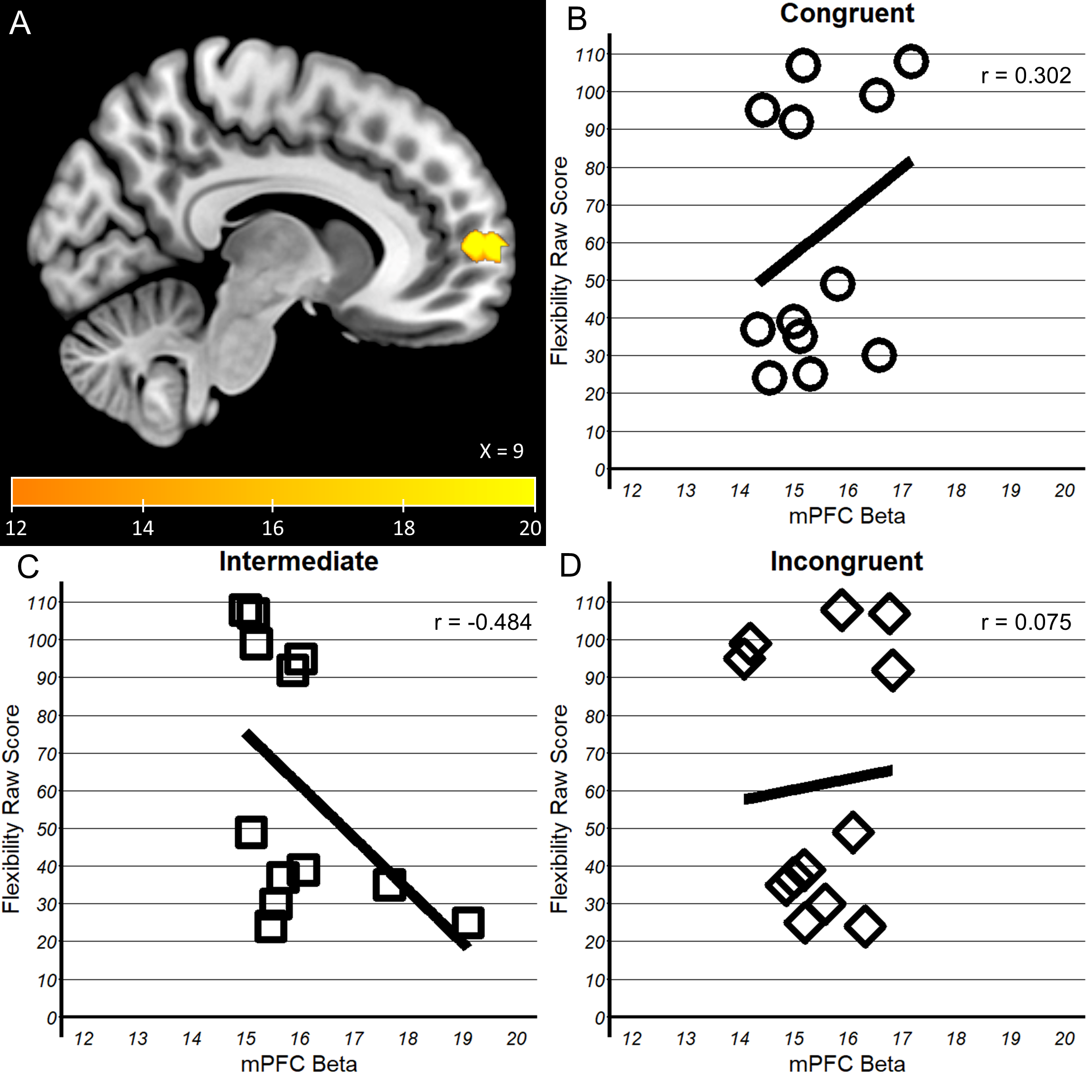
 SI Figure 3: Congruency differences in ASD children after controlling for age*

*Results of the Congruency (Congruent, Intermediate, Incongruent) X Flexibility with Age as a covariate in the ASD sample with the Associative Hits>Miss trials contrast. A significant cluster (A) was observed matching the cluster in the main text showing the same positive Congruent (B), negative Intermediate (C), and negligible Incongruent (D) relationships.*

*
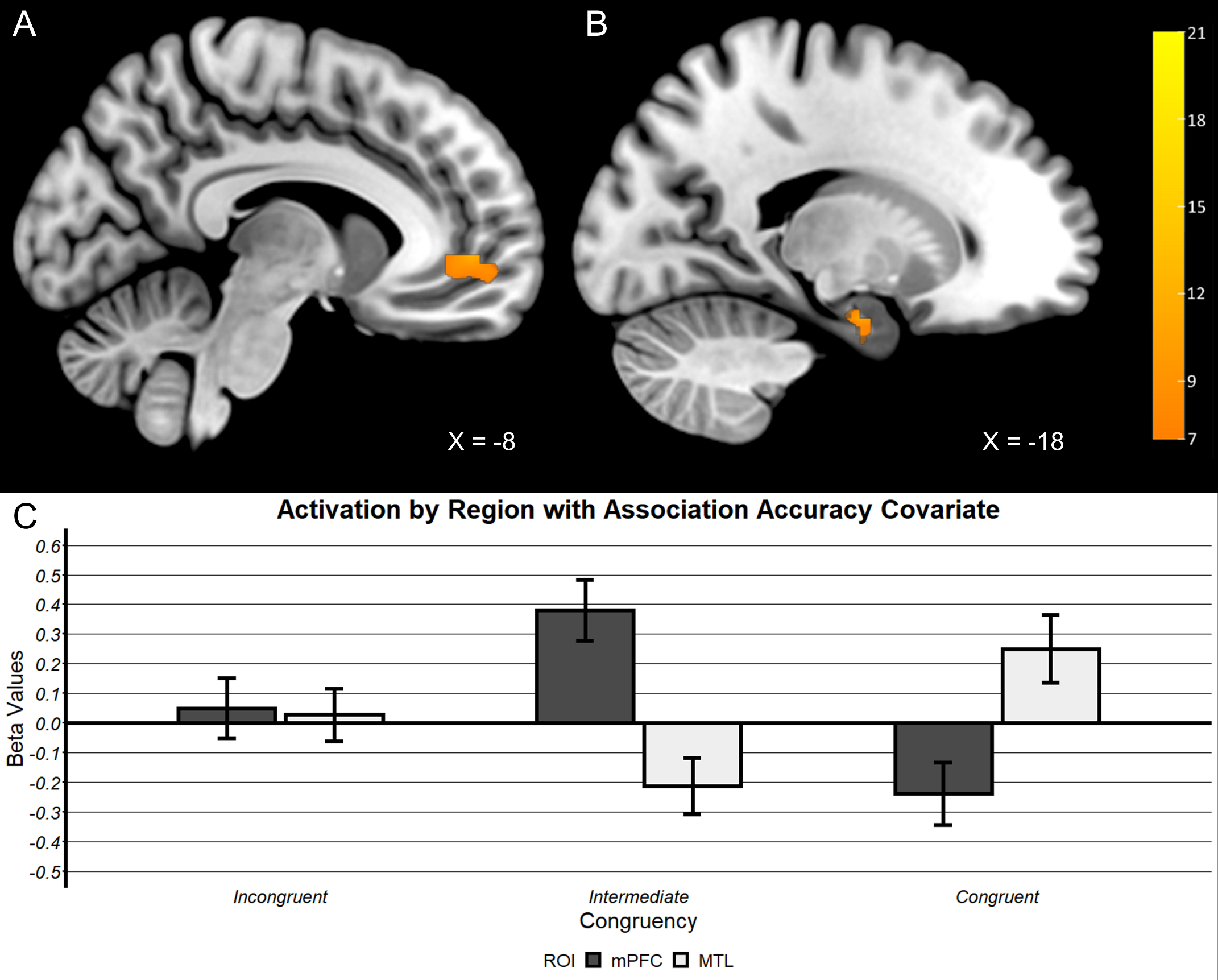
*Second, we wanted to determine whether the observed activations were due to individual variability in associative memory performance. The ANCOVA including the number of hit trials revealed similar clusters in TD children, mPFC (k = 62, peak = 6, 54, -6) and MTL (k = 24, peak = -30, -12, -21) as those with the ANOVA without hit trials (SI Figure 4). Similarly, children with ASD exhibited a Congruency X Flexibility interaction in the same cluster (k = 43 peak = 9, 63, 6) with the same relationship between flexibility and the three levels of congruency as was observed without the covariate (SI Figure 5). The similarities indicate that observed results did not depend on differences in memory performance.

*SI Figure 4: Congruency differences in TD children after controlling for the number of associative hits*

*
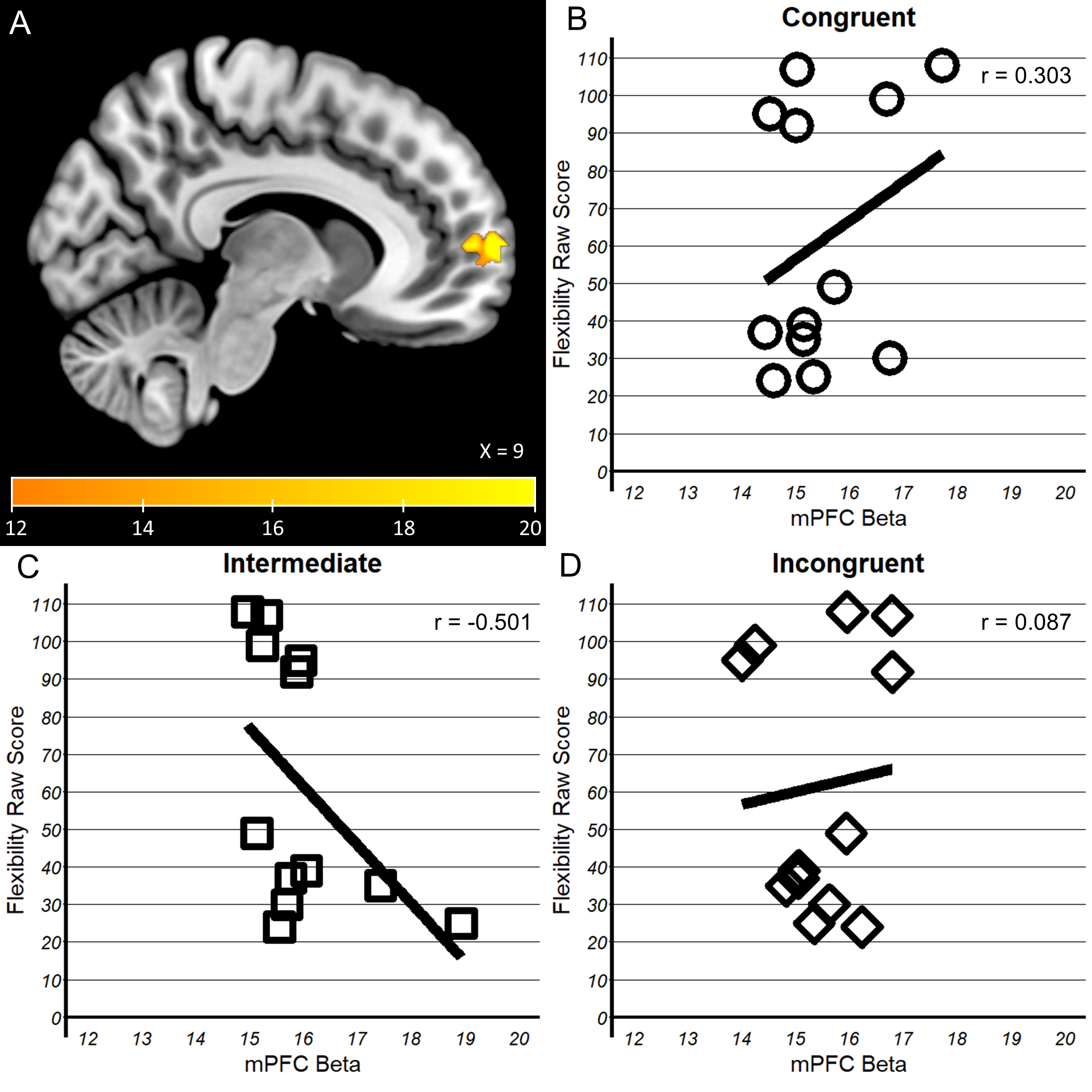
Results of the one-way ANCOVA by Congruency (Congruent, Intermediate, Incongruent) with number of associate hits as a covariate in the TD sample with the Associative Hits>Miss trials contrast, in the mPFC (A) and the left MTL mask (B); extracted beta values from each cluster are plotted in (C).*

*SI Figure 5: Congruency differences in ASD children after controlling for the number of associative hits*

*Results of the Congruency (Congruent, Intermediate, Incongruent) X Flexibility with Associate Hits as a covariate in the ASD sample with the Associative Hits>Miss trials contrast A significant cluster (A) was observed matching the cluster in the main text showing the same positive Congruent (B), negative Intermediate (C), and negligible Incongruent (D) relationships.*

Third, we wanted to determine whether differences were a function of time spent consciously deliberating on pairs, which resulted in significantly slower response times for Intermediate pairs and thus more time spent actively thinking about those pairs relative to Congruent and Incongruent. The ANCOVA resulted in similar clusters in TD children, mPFC (k = 206, peak = -3, 48, 0) and MTL (k = 9, peak = -15, -6, -27) as those with the ANOVA without response time (Figure 6). Similarly, children with ASD exhibited a Congruency X Flexibility interaction in the same cluster (k = 47 peak = 11, 63, 1) with the same relationship between flexibility and the three levels of congruency as was observed without response time as a covariate (Figure 7). The similarities indicate that observed results did not depend on encoding response time.

*
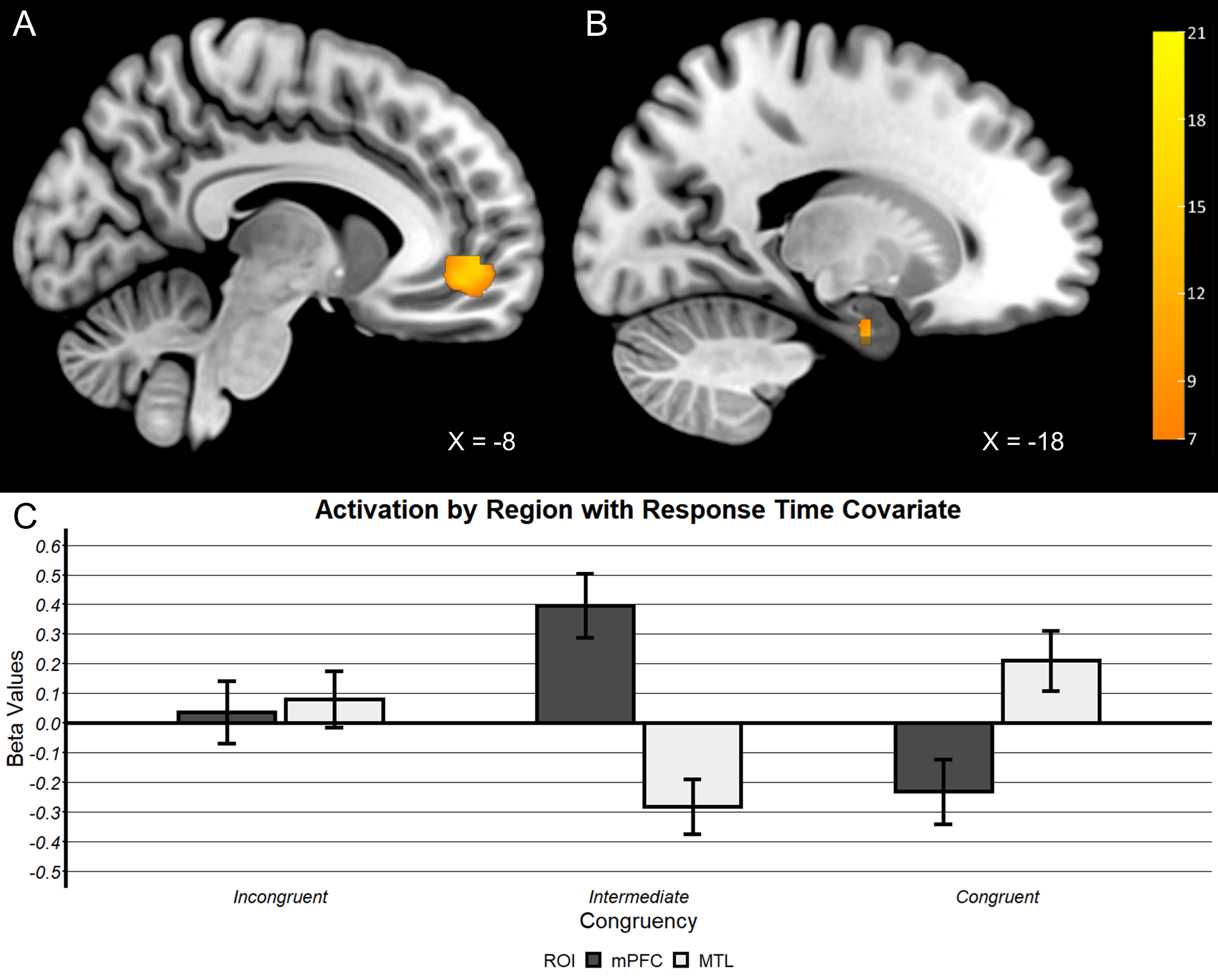
*

*SI Figure 6: Congruency differences in TD children after controlling for response time*

*Results of the one-way ANCOVA by Congruency (Congruent, Intermediate, Incongruent) with average Response Time as a covariate in the TD sample with the Associative Hits>Miss trials contrast, in the mPFC (A) and the left MTL mask (B); extracted beta values from each cluster are plotted in (C).*

*
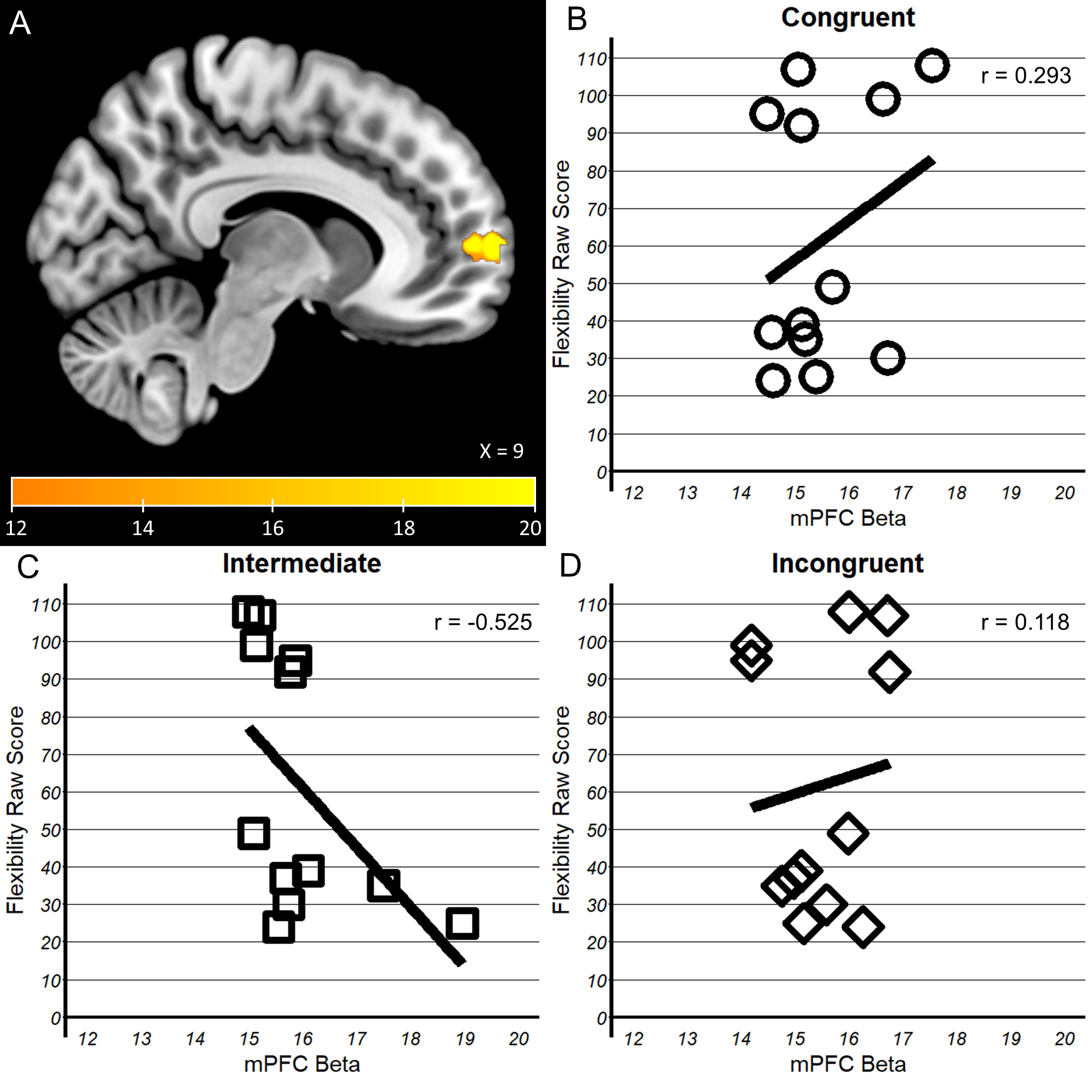
 SI Figure 7: Congruency differences in ASD children after controlling for response time*

*Results of the Congruency (Congruent, Intermediate, Incongruent) X Flexibility with Associate Hits as a covariate in the ASD sample with the Associative Hits>Miss trials contrast A significant cluster (A) was observed matching the cluster in the main text showing the same positive Congruent (B), negative Intermediate (C), and negligible Incongruent (D) relationships.*

Fourth, we wanted to determine whether the observed differences were an artifact of gender which was biased towards males (74%). The ANCOVA resulted in similar clusters, mPFC (k = 58, peak = -12, -12, -21) and MTL (k=8, peak = -3, -47, -2) as those with the ANOVA without gender (Figure 8). Similarly, children with ASD exhibited the Congruency X Flexibility interaction in the same cluster (k = 43 peak = 16, 63, 0) with the same relationship between flexibility and the three levels of congruency as was observed without the covariate (SI 9). The similarities indicate that observed results did not depend on gender differences.

*
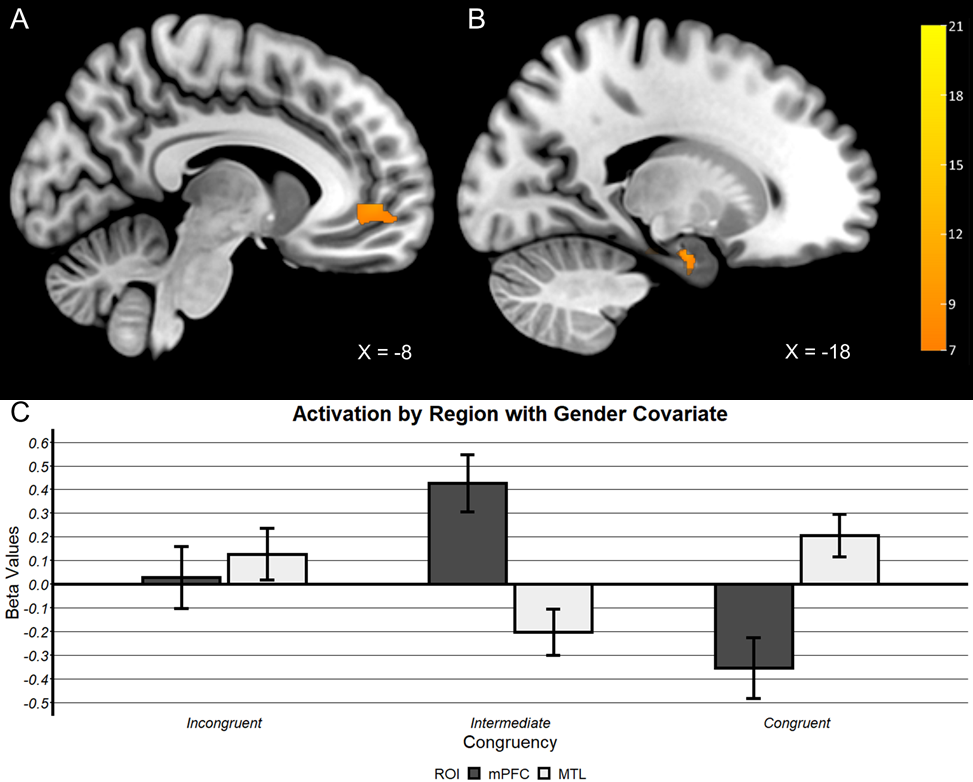
*

*SI Figure 8: Congruency differences in TD children after controlling for gender*

*Results of the one-way ANCOVA by Congruency (Congruent, Intermediate, Incongruent) with gender as a covariate in the TD sample with the Associative Hits>Miss trials contrast, in the mPFC (A) and the left MTL mask (B); extracted beta values from each cluster are plotted in (C).*

*
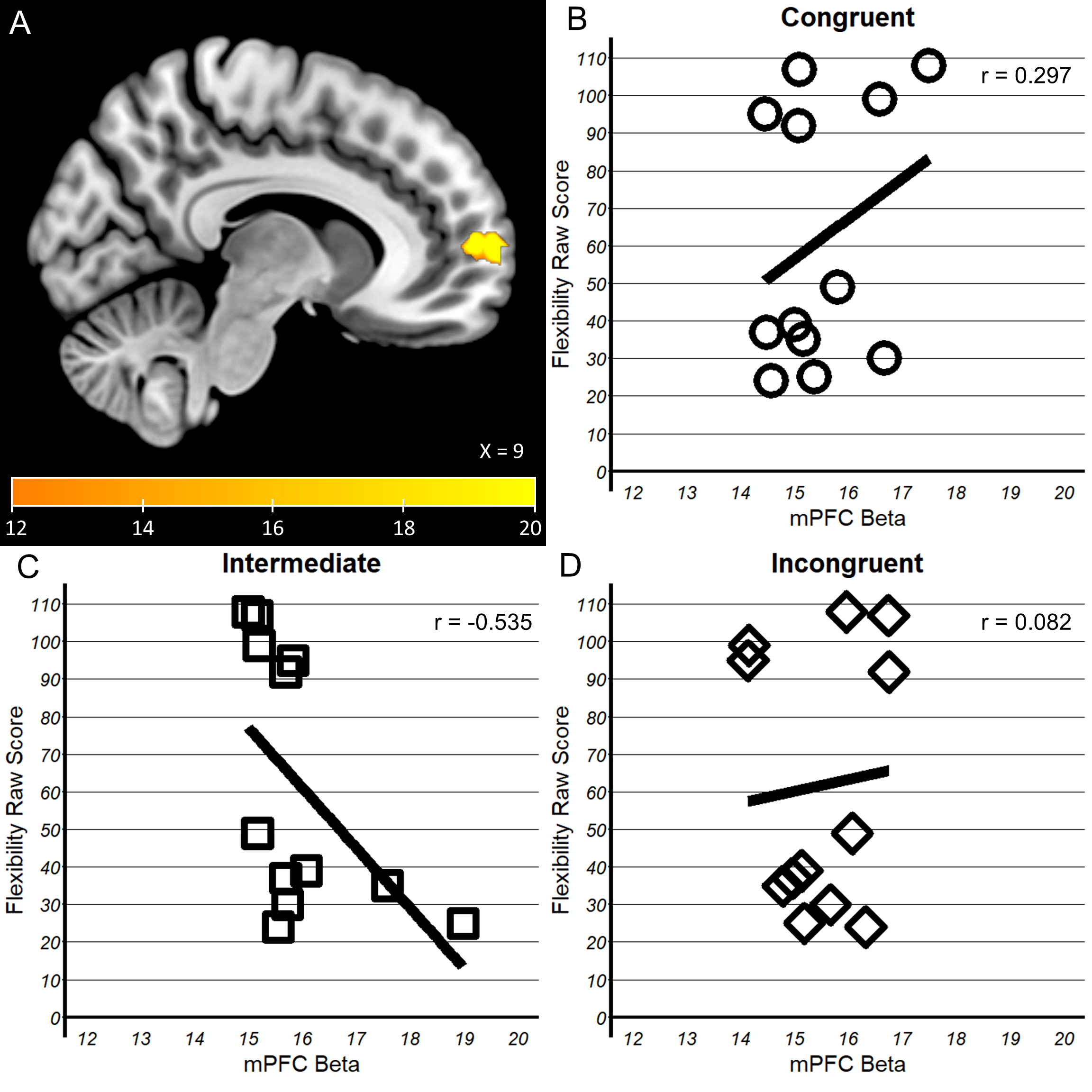
 SI Figure 9: Congruency differences in ASD children after controlling for gender*

*Results of the Congruency (Congruent, Intermediate, Incongruent) X Flexibility with Associate Hits as a covariate in the ASD sample with the Associative Hits>Miss trials contrast A significant cluster (A) was observed matching the cluster in the main text showing the same positive Congruent (B), negative Intermediate (C), and negligible Incongruent (D) relationships.*

In sum, controlling for age, gender, associative memory performance, and encoding response time, did not change the primary results reported in the main text, that mPFC and MTL activations were driven by a trade off between intermediate and congruent pairs in TD children and that mPFC activation for encoding successfully remembered object-scene pairs of intermediate congruency varied by the behavioral flexibility of children with ASD.
